# A protein vaccine of RBD integrated with immune evasion mutation shows broad protection against SARS-CoV-2

**DOI:** 10.1038/s41392-024-02007-8

**Published:** 2024-11-06

**Authors:** Ran An, Hao Yang, Cong Tang, Qianqian Li, Qing Huang, Haixuan Wang, Junbin Wang, Yanan Zhou, Yun Yang, Hongyu Chen, Wenhai Yu, Bai Li, Daoju Wu, Yong Zhang, Fangyu Luo, Wenqi Quan, Jingwen Xu, Dongdong Lin, Xiaoming Liang, Yuhuan Yan, Longhai Yuan, Xuena Du, Yuxia Yuan, Yanwen Li, Qiangming Sun, Youchun Wang, Shuaiyao Lu

**Affiliations:** 1 Institute of Medical Biology, Chinese Academy of Medical Sciences and Peking Union Medical College, Kunming, China; 2 Key Laboratory of Pathogen Infection Prevention and Control (Peking Union Medical College), Ministry of Education, Beijing, China; 3 State Key Laboratory of Respiratory Health and Multimorbidity, Beijing, China; 4 Yunnan Provincial Key Laboratory of Vector-borne Diseases Control and Research, Kunming, China

**Keywords:** Infectious diseases, Vaccines

## Abstract

Variants of severe acute respiratory syndrome coronavirus 2 (SARS-CoV-2) continue to emerge and evade immunity, resulting in breakthrough infections in vaccinated populations. There is an urgent need for the development of vaccines with broad protective effects. In this study, we selected hotspot mutations in the receptor-binding domain (RBD) that contribute to immune escape properties and integrated them into the original RBD protein to obtain a complex RBD protein (cRBD), and we found cRBDs have broad protective effects against SARS-CoV-2 variants. Three cRBDs were designed in our study. Compared with the BA.1 RBD protein, the cRBDs induced the production of higher levels of broader-spectrum neutralizing antibodies, suggesting stronger and broader protective efficacy. In viral challenge experiments, cRBDs were more effective than BA.1 RBD in attenuating lung pathologic injury. Among the three constructs, cRBD3 showed optimal broad-spectrum and protective effects and is a promising candidate for a broad-spectrum SARS-CoV-2 vaccine. In conclusion, immunization with cRBDs triggered immunity against a wide range of variants, including those that emerged after we had completed designing the cRBDs. This study preliminarily explores and validates the feasibility of incorporating hotspot mutations that contribute to immune evasion into the RBD to expand the activity spectrum of antigen-induced antibodies.

## Introduction

Coronavirus disease 2019 (COVID-19) caused by SARS-CoV-2 has rapidly triggered a global public health emergency. According to the WHO, as of January 31, 2024, there were more than 770 million confirmed cases, with more than 77 million confirmed deaths.^1^ SARS-CoV-2 is an enveloped single-stranded positive-sense RNA virus, which is identical to Severe Acute Respiratory Syndrome Coronavirus and Middle East Respiratory Syndrome Coronavirus, belonging to the subgenus of *Sarbecovirus*. The SARS-CoV-2 genome is approximately 30 kb, and the 3′-UTR contains four open reading frames that encode for the generation of structural proteins, including Nucleocapsid, Spike (S), Membrane, and Envelope proteins.^2^ These structural proteins are responsible for viral particle assembly and are involved in suppressing the host immune response. S is a structural protein of SARS-CoV-2, which composed of two subunits, S1 and S2, and is responsible for target recognition, binding and cell entry of SARS-CoV-2.^3^ The S1 subunit comprises an N-terminal domain (NTD) and a C-terminal RBD. RBD is responsible for recognizing the angiotensin-converting enzyme 2 receptor (ACE2), thereby mediating SARS-CoV-2 entry.^4^ The RBD is immunogenic and is the primary target of neutralizing antibodies (nAbs) as well as a favorable antigen for vaccine development.^5^

Continued extensive spread of the virus has resulted in antigenic drift of SARS-CoV-2 genome. Over time, the high mutation rate of the SARS-CoV-2 genome has resulted in the continual generation of variants, resulting in changes of transmissibility, pathogenicity, and immunological resistance of COVID-19 regionally and globally.^6^ And SARS-CoV-2 variants such as B.1.1.7、B.1.315、P.1、B.1.617.2 and BA.1.1.529 have been identified as variants of concern (VOCs), because of the higher transmissibility, disease severity, and hospitalization rates.^7^ SARS-CoV-2 variants carrying certain mutation sites such as E484K show resistance to nAbs produced by natural infection or vaccination, leading to a reduction in the effectiveness of most immunotherapies, that is particularly pronounced in the Omicron variant. Vaccination is the most effective method for reducing the incidence and mortality of COVID-19.^7^ The vaccines have weakened their effectiveness against newly emerging variants because of their mutations in the S protein, especially in the RBD protein region, which is the target of existing vaccine.^8–11^ The S protein of the Omicron variant carries more than 30 mutations, half of which are located in the RBD region, which results in poor neutralizing activity against this variant even in sera obtained from patients recovering from COVID-19 or from people receiving booster vaccines.^12,13^ One study reported that the neutralizing effects of serum against BQ.1, BQ.1.1, XBB, and XBB.1 viruses were significantly impaired in vaccinated and infected individuals who received the WA.1/BA.5 bivalent mRNA vaccine.^14^ Titers against BQ and XBB variants were reduced by 13- to 81-fold and 66- to 155-fold, respectively.^14^ In conclusion, immunization with proteins of emerging strains as antigens has a short protective effect against future emerging variants. At the same time, the strategy of continuously updating vaccine antigens for supplementary immunization always lags behind the rate of virus variation. Thus, there is an urgent need to develop universal vaccines that more robustly induce the production of nAbs to combat emerging variants with unique immune escape abilities.

The conventional approach to developing broad-spectrum vaccines is to mix the antigens of new emerging variants to prepare a polyvalent vaccine, which is undoubtedly time-consuming and expensive. Currently, a common approach to the development of broad-spectrum SARS-CoV-2 vaccine antigens is to combine antigens from different variants into hybridized multimers.^15–17^ Yu Liang et al. developed a trimer RBD protein vaccine based on the prototype, beta, and kappa strains, which, induced a broad sera neutralization of SARS-CoV-2 variants.^17^ A more desirable goal would be to develop a vaccine with a single antigen that provides protection against all variants of SARS-CoV-2. Based on research of multimeric RBD protein vaccine, we speculate whether broad protection against all variants of could be achieved if a single RBD protein contained immune evasion mutations from all variants.

Here, we chose the RBD as the immunogen and designed a novel RBD sequence (cRBD) by introducing characteristic mutation sites from each variant. The protein was designed at the time of the emergence of BA.5 variants and contains mutation sites in VOCs associated with immune evasion that have been reported previously. A total of three cRBD constructs were designed and expressed, the BA.1 RBD protein was used as a comparative control, and a comprehensive immunological evaluation of the RBD antigen designed and synthesized via this strategy was conducted. In Balb/c mice, cRBD-based vaccines induced neutralizing activity against VOCs including the recently prevalent JN.1, EG.5.1, and XBB. And the vaccine showed protective efficacy against EG.5.1 in Balb/c and K18-hACE2 mice model. The results preliminarily confirmed the feasibility of developing a broad-spectrum vaccine by integrating immune evasion mutation sites, providing some experience for the development of broad-spectrum vaccines of SARS-CoV-2.

## Results

### Design and preparation of the cRBD vaccine

**Fig. 1:**
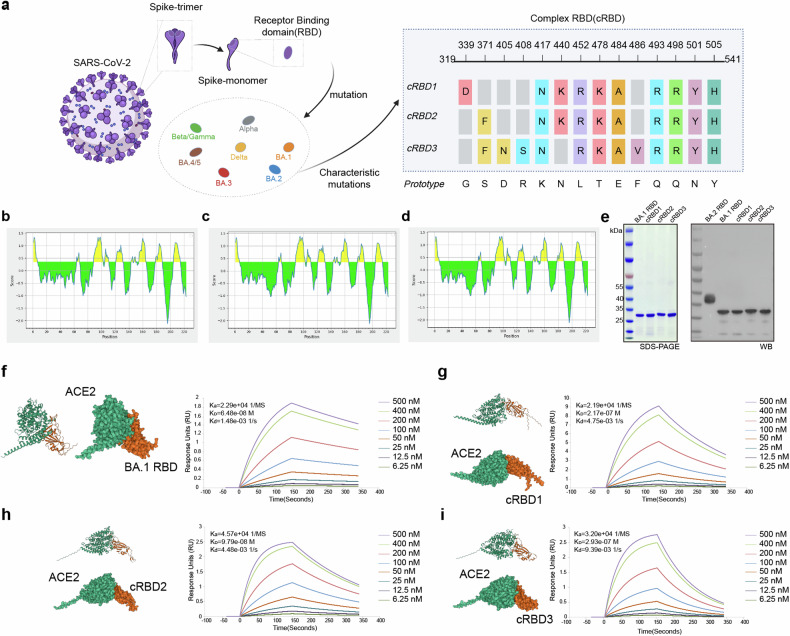
Design and preparation of the cRBD vaccine. **a** Schematic diagram of cRBD sequence design (adapted from “Multiple Sequence Alignment (Protein)”, by BioRender.com (2024). Retrieved from https://app.biorender.com/biorender-templates). The RBD region (319-541) on the S protein of the prototype strain was selected and specific mutation sites were added to obtain cRBD1-3. **b**–**d** Results of B-cell linear antigenic epitopes prediction for cRBD1-3. **e** SDS‒PAGE and Western-blot results of cRBD and BA.1 RBD proteins expressed by *E. coli*. The BA.2 RBD protein was a positive control in the Western-blot assay. As a eukaryote-expressed protein, it migrates as an ~36.55 kDa band in SDS‒PAGE under reducing conditions due to glycosylation. **f**–**i** Surface and cartoon representation of the interaction of hACE2 with BA.1 RBD and cRBD1-3. hACE2 is displayed in green, and cRBD or BA.1 RBD in orange. Surface plasmon resonance recorded the profile of a real-time affinity of hACE2 to BA.1 RBD and cRBD1-3

The B-cell linear antibody epitopes of the RBD proteins of some variants were analysed using IEDB.^18^ We found that the overall antigenic peptides of each RBD region appeared to be relatively similar. However, the antigenicity of some peptides seems to be different, indicating that some amino acid mutations may change the antigenicity of the whole peptide. For example, omicron variants (BA.1, BA.2, BA.3, and BA.4/5) showed an overall decrease in the antigenicity of RBD tail peptides (Supplementary Fig. 1). The key amino acid mutations in the RBD often cause immune escape of the virus. Mutations at E484 and F456 lead to a loss of neutralizing potency of sera from infected or immunized individuals. Moreover, mutations in G339, S371, K417, F486, L452, Q493, N501, and other sites may also reduce the antibody neutralization capacity. In addition, mutations in some of these sites directly reduce the receptor binding capacity of the RBD.^19–22^ At the same time, some conserved peptides are also critical for the ability of RBD to induce neutralizing antibodies.^23^ Here RBD sequences were designed according to the immune escape characteristics of each mutant RBD region and the antigenic characteristics of the RBD region of S protein (Fig. 1a). The antigen prediction results showed that the designed constructs did not change the overall antigen characteristics of the RBD (Fig. 1b–d).

It has been reported that RBD protein obtained using prokaryotic expression system can induce an immune response after immunization in animals.^24^ Thus, we use the prokaryotic expression system to obtain the protein (the detailed steps are described in the “Methods” section). Sodium dodecyl sulfate-polyacrylamide gel electrophoresis (SDS‒PAGE) of the recombinant RBD proteins revealed a single band with a molecular weight of approximately 30 kDa (Fig. 1e), which is consistent with the theoretical value, indicating protein integrity and purity. Western-Blots verified that the purified proteins were indeed RBD proteins (Fig. 1e). Further, the structure of BA.1 RBD and cRBD proteins as well as their binding sites to human ACE2 (hACE2) were predicted using AlphaFold3 (Fig. 1f–i), and the results showed that the binding sites of the modified RBD protein with ACE2 have changed (Supplementary Fig. 2). Using surface plasmon resonance (SPR), we confirmed the binding and observed effective affinity between them (Fig. 1f–i). This represents that the proteins we obtained using the prokaryotic expression system have the correct conformation as well as biological activities. Moreover, the results of SPR showed that hACE2 had the highest affinity for BA.1 RBD and the lowest for cRBD3. The changes in the binding site and affinity of the modified RBD proteins for ACE2 might be responsible for the changes in the immune responses they induced. The adjuvant AddaS03 was mixed with protein to prepare a vaccine for subsequent experiments.

### cRBDs induce strong immune responses in BALB/c mice

**Fig. 2:**
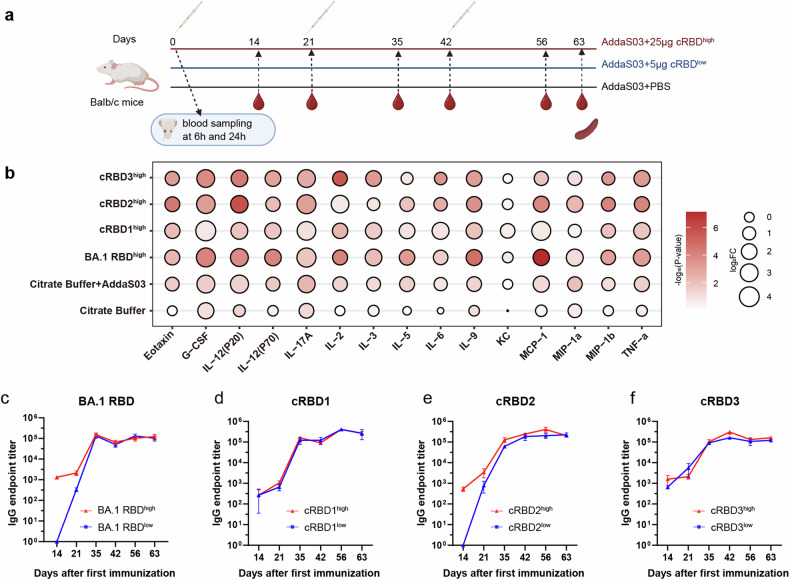
cRBDs induced strong immune responses in BALB/c mice. **a** The timeline of vaccine immunization and sampling (created with BioRender.com). The serum used for cytokine detection was collected at 6 h and 24 h after the first injection. Final blood collection was performed at 14 and 21 days (Days 14 (*n* = 3), 21 (*n* = 3), 35 (*n* = 5), 42 (*n* = 5), 56 (*n* = 5), and 63 (*n* = 5) in (**a**)) after each immunization to collect serum for detection of vaccine-induced production of binding and neutralizing antibodies. Spleens were collected simultaneously 21 days after the third injection (day 63) for an ELISPOT assay. **b** Fold change in the levels of inflammation-related cytokines in the serum at 6 h and 24 h after immunization (*n* = 5). The color of the circle represents the *P*-value. The smaller the *P* value is, the redder the color. The size of the circle represents the fold change (FC). FC = cytokine level at 6 h after immunization /cytokine level at 24 h after immunization. The greater the FC is, the larger the circle. **c**–**f** BA.2 RBD-specific binding antibody levels at each time point after immunization in each group. Data are presented as mean ± SD

As shown in Fig. 2a, a three-injection strategy was used with a 21-day interval between each immunization, and for each injection, two different doses, namely, a low dose (5 μg/dose) and a high dose (25 µg/dose), were applied. To confirm whether the immune response could be activated after vaccination, we measured the expression levels of cytokines and chemokines in the serum at 6 h and 24 h (Fig. 2b). In addition to establishing the vaccine-immunized group, we also established an adjuvant group and a buffer group as control groups. Compared with those in the buffer group, the levels of many cytokines in the adjuvant and vaccine groups increased more obviously at 6 h post-injection than at 24 h post-injection. For example, IL-5, which is a critical cytokine for B-cell differentiation to antibody-secreting plasma cells in mice,^25^ and IL-6, which is important for B-cell proliferation and isotype switching.^26^ Furthermore, the production of MCP-1, MIP-1a, MIP-1b, and TNF-α, which are key chemokines for antigen-presenting cell activation and migration, was also induced at 6 h compared with 24 h.^26,27^ No such change was found in the citrate buffer injection group. These findings suggest that the vaccine induces an immune response and that this response is partly induced by the adjuvant.

Considering that most of the currently common variants evolved from BA.2, we measured BA.2 RBD-binding antibody levels on days 14 and 21 after each injection. On day 14 after the first immunization, BA.2 RBD-binding antibodies were detected in all groups except the cRBD2 low-dose and BA.1 RBD low-dose groups, and they were detected in all groups on day 21. Moreover, there were no differences between the high- and low-dose in cRBD1 and cRBD3 groups, while the cRBD2 and BA.1 RBD groups showed some differences only before the second immunization (higher levels of binding antibodies in the high-dose group). As the immunization process progressed, there was an overall trend toward increasing binding antibody levels. Antibody titers at the end of the immunization process (63 d) were ~1 × 10^5^ in all groups, which indicates that all proteins have good immunogenicity.

### cBRD3 elicits the production of antibodies with broadly neutralizing activity against SARS-CoV-2 variants

**Fig. 3:**
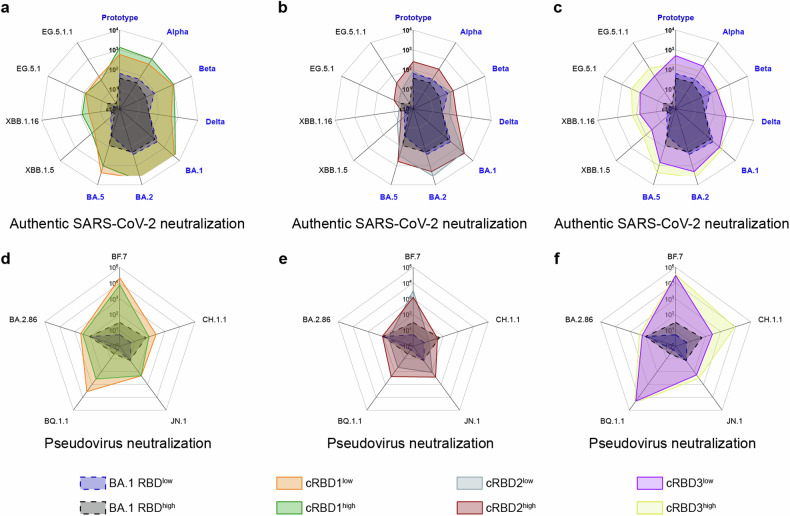
Neutralization activity of cRBD-induced antibodies against each strain. Geometric mean titer (GMT) of serum nAbs for authentic virus (**a**–**c**) (*n* = 5) and pseudovirus (**d**–**f**) (*n* = 3) 21 days after the third immunization (day 63 in Fig. 2a) for each group. Specific GMT values are presented in Supplementary Figs. 3–4. For ease of visualization, the cRBD1-3 results are plotted separately, and the results for the BA.1 RBD group are added as a control in each plot

It has been reported that higher levels of nAbs are associated with lower chances of immune evasion.^28^ Therefore, nAbs titers against different SARS-CoV-2 variants are key indicators for assessing the breadth of the protective capacity of a vaccine. We investigated whether the designed proteins could elicit a broadly neutralizing antibody response against a wide range of variants by authentic and pseudoviral neutralization experiments and with BA.1 RBD as a control. The immunization procedure was the same as that described above (Fig. 2a), but blood was collected only on days 35, 42, 56, and 63. We detected the variants that appeared before the sequence design, including the prototype, alpha, beta, delta, BA.1, BA.2, and BA.5, and the strains that appeared after the sequence design, including XBB.1.15, XBB.1.16, EG.5.1, and EG.5.1.1 (Supplementary Fig. 3, Fig. 3a–c). In addition, some strains lacking available authentic viruses were complemented by pseudoviruses, including BF.7, CH.1.1, BQ.1.1, BA.2.86, and JN.1 (Supplementary Fig. 4, Fig. 3d–f).

Consistent with the results of the binding antibody assay, the level of nAbs against each strain increased as the immunization period progressed. cRBD1 induced a more rapid and stronger immune response, inducing the production of higher levels of nAbs at 14 days after the first immunization than in the other groups, regardless of the strain (Supplementary Fig. 3). Serum nAbs levels in mice immunized with BA.1 RBD were much lower than those in the mice immunized with the cRBD proteins, even that against BA.1 (Fig. 3a–c). nAbs against all tested strains were detected in cRBD-immunized mouse sera, even against the recently emerged BA.2.86 and JN.1 strains. However, some variants, such as CH.1.1, XBB.1.15, XBB.1.16, EG.5.1, and BA.2.86, were significantly less sensitive to neutralization by immunizing serum (Fig. 3a–c). Moreover, the levels of nAbs against variants were different among the three experimental groups. The neutralizing ability of the nAbs whose production was induced in response to each of the three constructs against the JN.1 variant was similar, but compared to the other groups, the cRBD3 high-dose group showed superior neutralizing activity against the BA.2.86 variant. Serum from cRBD2-immunized mice showed a more pronounced decrease in neutralizing ability against the XBB.1.5, XBB.1.16, EG.5.1, and EG.5.1.1 variants. Compared with serum from mice immunized with cRBD1, serum from mice immunized with cRBD3 had a slightly reduced ability to neutralize variants that emerged before the construct was designed, but its ability to neutralize new variants that emerged after the construct was designed was greater than that of serum from mice immunized with cRBD1. In general, cRBD3 induces a broader neutralization response. In addition, levels of nAbs against multiple variants could still be detected in the serum of mice 18 weeks or longer after completion of immunization, and antibody levels were relatively higher in the cRBD-immunized group, suggesting that cRBD-based vaccine induces long-lasting protection in mice after immunization (Supplementary Fig. 5).

**Fig. 4:**
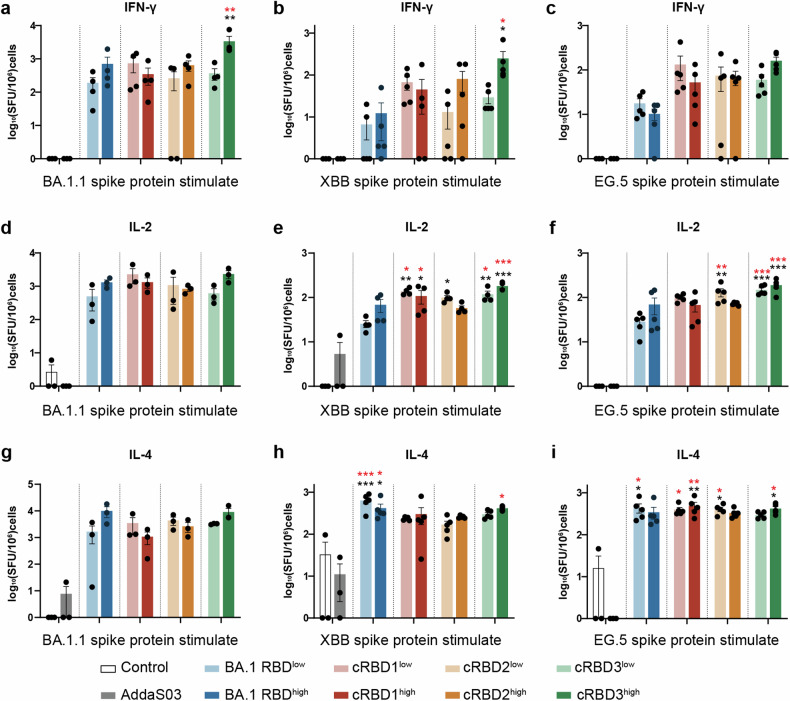
Statistical plots of the ELISPOT results. Three stimulants (BA.1.1, XBB, and EG.5 S protein) were used for detection. **a**–**c** The number of IFN-γ secreting cells in each group after stimulated (*n* = 3 in Control and AddaS03 group, *n* = 5 in other vaccinated groups). **d**–**f** The number of IL-2 secreting cells in each group after stimulated (*n* = 3 in Control and AddaS03 group, *n* = 3/4/5 in other vaccinated groups. **g**–**i** The number of IL-4 secreting cells in each groups after stimulated (*n* = 3 in Control and AddaS03 group, *n* = 3/5/5 in other vaccinated groups). The data are expressed as means ± SD. Statistical analysis was conducted using one-way ANOVA and Tukey’s multiple comparison test. The significance of differences between the experimental group and the unimmunized control group is indicated by black asterisks, the significance of differences between the experimental group and the adjuvant group is indicated by red asterisks, and no marker indicates no significance. *P < 0.05, **P < 0.01, ***P < 0.005

To explore the ability of cRBD to induce cellular immunity, we collected the spleens of mice that had completed the full immunization schedule and isolated the lymphocytes. ELISPOTs were performed using the S protein of the BA.1.1, XBB, and EG.5 variants as stimulants (Fig. 4, Supplementary Fig. 6-8). Compared to the adjuvant control group, lymphocytes from mice immunized with either BA.1 RBD or cRBD proteins showed higher IFN-γ, IL-2, and IL-4 secretion than those from unimmunized or adjuvant-immunized animals. It suggests that the vaccine-immunized group induced specific T-cell responses to S proteins of BA.1.1, XBB, and EG.5. However, there was no significant difference between the BA.1 RBD- and cRBD-immunized groups, suggesting similar cellular immunity. The results showed that cRBD immunization could induce specific cellular immunity against XBB, EG.5, and BA.1.1 but not to a greater extent than BA.1 RBD.

### cRBDs protect BALB/c mice from challenge with live SARS-CoV-2

**Fig. 5:**
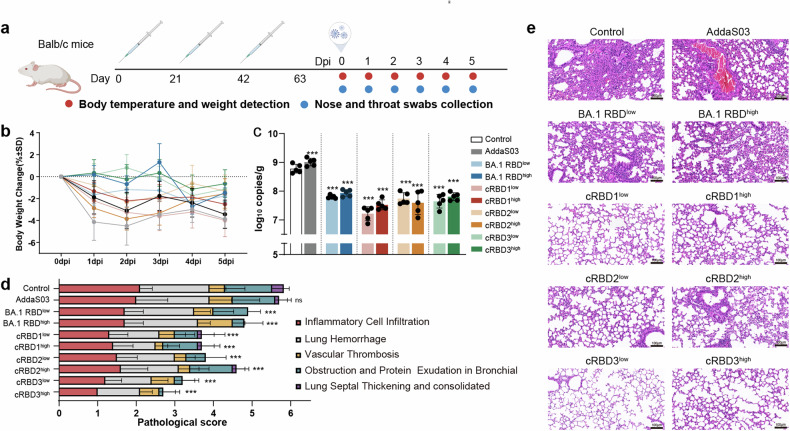
Results of EG.5.1 variant challenge in Balb/c mice after immunization. **a** Schematic illustration of the timeline of BALB/c mice immunization and challenge with SARS-CoV-2 (created with BioRender.com). Mice that were not immunized were used as controls. The challenge experiment was performed 21 days after the third immunization. **b** Body weight changes after infection in each group (*n* = 10). **c** Viral gRNA levels in the lung at 5 days post-infection (dpi) in each group (*n* = 5). **d** Histogram of mouse lung pathology scores in each group (*n* = 5). **e** Histopathological examination of mice lung tissues. Scale bar, 100 μm. The data are expressed as means ± SD. Results of each group were compared with the control group in c and d. Statistical analysis was conducted using one-way ANOVA and Tukey’s multiple comparison test for bar graphs. ***P < 0.005. ns not significant

Mice were immunized three times on days 0, 21, and 42, infected with SARS-CoV-2 on day 63, and dissected 5 days after challenge. Nasopharyngeal swabs and body temperature and weight data were collected daily during infection (Fig. 5a). After challenge with the EG.5.1 strain, the weights of the mice in all the groups tended to decrease, and the weight loss was the lowest in the cRBD3 high- and low-dose groups. Weight loss was most obvious in the control and adjuvant groups, except for the cRBD2 group (Fig. 5b). The nasopharyngeal swab loads of the control group and the vaccine group fluctuated greatly with time, but the swab load of some vaccine groups was still lower than that of the control group (Supplementary Fig. 9b, c). Moreover, the cRBD vaccine candidates significantly reduced the viral loads in the lungs of the infected mice (P < 0.05) (Fig. 5c). The pathological changes in the lungs of mice infected with the virus mainly manifested as pulmonary hemorrhage, inflammatory cell infiltration, vascular thrombosis, bronchial obstruction, and protein exudation (Fig. 5d, e). The degree of lung injury in the vaccine group was significantly lower than that in the control group, and the cRBD3 vaccine yielded the most robust lung protection (Fig. 5d, e). Although the BA.1 RBD was similar to the cRBDs in terms of ability to reduce the viral load, it was associated with worse lung pathology (Fig. 5d, e).

We also challenged the mice with the BA.5 variant after administering the cRBD1 vaccine and challenged mice immunized with the cRBD2 and cRBD3 vaccines with the XBB.1.5 variant (Supplementary Figs. 10–12). In the two challenge experiments, cRBD also showed great protective efficacy. The serum of mice immunized with cRBD showed low neutralizing activity against the strains that used in the challenge test (Fig. 3), and the level of neutralizing antibodies was positively correlated with the protective efficacy of the vaccine.^28^ Therefore, it can be speculated that the cRBD-based vaccine has a protective effect on all variants involved in neutralization test. The cRBD3 vaccine showed a broad-spectrum inhibitory effect against XBB.1.5 and EG.5.1 virus infection.

### cRBDs protect K18-hACE2 mice from challenge with SARS-CoV-2

**Fig. 6:**
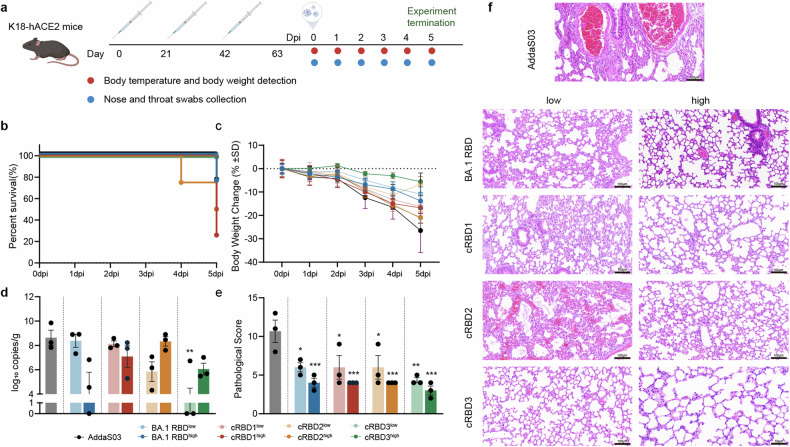
Results of EG.5.1 variant challenge in K18-hACE2 mice after immunization. **a** Schematic illustration of the timeline of BALB/c mice immunization and challenge with SARS-CoV-2 (created with BioRender.com). **b** Changes in survival rate of mice in each group within 5 days of infection. **c** Body weight changes after infection in each group (*n* = 3). **d** Viral gRNA levels in the lung at 5 dpi in each group (*n* = 3). **e** Histogram of mouse lung pathology scores in each group (*n* = 3). **f** Histopathological examination of mice lung tissues. AddaS03 as adjuvant control. The data are expressed as means ± SD. Results of each group were compared with the AddaS03 group in d and e. Statistical analysis was conducted using one-way ANOVA and Tukey’s multiple comparison test for bar graphs. *P < 0.05, **P < 0.01, ***P < 0.005

In order to more realistically evaluate the protective effect of the cRBD vaccine, we evaluated the protective efficacy of the cRBD-based vaccine against SARS-CoV-2 using the K18-hACE2 model mice.^29^ As in the previous procedure, mice were immunized three times, and two weeks later the EG.5.1 attack experiment was performed (Fig. 6a). We detected similar levels of BA.2 RBD-binding antibodies in K18-hACE2 mice as in Balb/c mice during immunization, but lower levels of nAb and memory immune cells were induced in K18-hACE2 mice after vaccine immunization (Supplementary Fig. 13a–e). We measured the body temperature and body weight as well as the viral load of nasopharyngeal swabs of each group of mice during infection period, but no significant differences were observed between groups. (Supplementary Fig. 13f–h).

Mice were dissected 5 days after infection. Death occurred in some immunized groups during infection, with mice in the cRBD1 low-dose group having the lowest survival rate at the end of the attack (Fig. 6b). The body weight of mice in all groups showed a decreasing trend after infection, with the greatest decrease in the adjuvant group and the lowest decrease in the cRBD3 high-dose group (Fig. 6c). In addition, cRBD-based vaccine immunization significantly reduced the viral load in the lungs of mice, with a significant reduction in viral load observed in the cRBD3 low-dose immunization group, suggesting that it exerted the most effective effect in up-suppressing viral replication (Fig. 6d). In addition, vaccine immunization significantly reduced lung pathological damage in all groups of mice, with the most significant reduction in lung pathological damage in the cRBD3-immunized group, suggesting that cRBD3 may play the most potent lung-protecting role in the challenge experiments (Fig. 6e, f). Therefore, we can speculate that the vaccine based on cRBD3 protein may exert the most obvious protective effect in the EG.5.1 challenge experiment.

### Differences in the B-cell receptor repertoire induced by cRBDs

To further explore the reasons for the broad-spectrum differences after vaccination from the molecular aspect, we collected whole blood of mice in the cRBD high-dose group 21 days after completing three immunizations for transcriptome and B-cell receptor (BCR) sequencing. Another set of untreated mice was used as a negative control (NC) group.

In terms of the transcriptome, Gene Ontology (GO) enrichment of the differentially expressed genes (DEGs) (|log_2_FC| > 2, *P* < 0.05) was performed between the cRBD groups and NC group, and each group exhibited enrichment for immune- or inflammation-related pathways (Supplementary Fig. 14a–c). This finding suggested that immunization with these vaccines affects the immune system. Using the Reactome database,^30^ we identified genes related to innate and adaptive immunity pathways and analysed their expression among the groups. The expression of most of the IFN-γ pathway genes increased in the cRBD1 immunization group, followed by the cRBD3 immunization group, while the expression of most of the IFN-γ pathway genes tended to decrease in the cRBD2 immunization group (Supplementary Fig. 14d). The expression of genes complexed with MHCI and MHCII, which promote antigen presentation to CD4 and CD8 molecules, were also examined in each experimental group. CD8^+^T cells mainly participate in cellular immunity, while CD4^+^T cells mainly promote the proliferation and differentiation of B cells and participate in humoral immunity. Similar to that of IFN-γ, most of the gene expression in the cRBD1 group was induced (Supplementary Fig. 14e, f). This finding suggested that cRBD1 immunization may induce stronger humoral and cellular immunity. In our previous experiments, we confirmed that cRBD1 induces faster and stronger humoral immunity after immunization, as determined by serial detection of nAbs, and that higher levels of antibodies can be detected as early as 14 days after a single immunization (Supplementary Fig. 3).

**Fig. 7:**
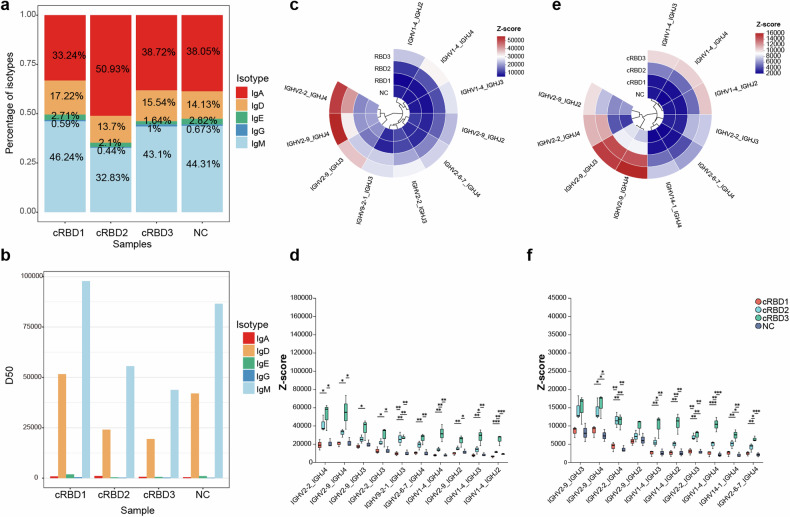
Analysis of BCR sequencing results. **a** Cumulative histogram of the proportion of each isoform of IGH in each group. **b** Statistical plot of the diversity of IGH isoforms in each group, using D50 as an indicator, with larger D50 values indicating greater diversity of IGH in the samples. **c** Expression levels of top-10 used V-J pairs in IgM. **d** Statistical analysis of the expression levels of the top 10 V-J genes associated with IgM in each group. **e** Expression levels of the top 10 V-J pairs used for IgD. **f** Statistical analysis of the expression levels of the top 10 V-J genes associated with IgD in each group. Statistical analysis was conducted using multiple tests and corrected for FDR, with Scheffe post hoc tests. *P < 0.05, **P < 0.01, ***P < 0.005. ns no significant. *n* = 3 in each group

For BCR sequencing, we first compared the abundances of different immunoglobulin types in the NC and vaccine-immunized groups, and the abundances of IgM and IgD were greater in all groups (Fig. 7a). Moreover, the diversity of IgM and IgD was much greater than that of the other immunoglobulins (Fig. 7b). Therefore, we focused on IgD and IgM and further assessed the use of V-J genes in the IgD and IgM heavy chain proteins of each group. The abundance of the top 10 V-J gene pairs with the highest frequency of use in each group for the IgM and IgD isoforms is shown in Fig. 7c and, e. Among the IgM subtypes, v2-2_J4, v2-9_J4, v2-9_J3, and v1-4_J4 showed abnormally high abundances in the cRBD3-immunized group (Fig. 7c). Further statistical analysis revealed that among the top ten most abundant V-J pairs, those in the cRBD3 group had significantly greater abundances than did those in the other groups (Fig. 7d). Similarly, for IgD, we observed the same difference in V-J usage (Fig. 7f). We hypothesized that this change in antibody abundance may be the reason why cRBD3 induced a better broad-spectrum response.

## Discussion

The emergence of variants was inevitable due to widespread SARS-CoV-2 infection in a wide range of populations and susceptible animals, leading to widespread escape from most antibodies and vaccine responses.^31–34^ Notably, BA.4/5 or XBB.1.5 variant infection produced little or no neutralizing activity against EG.5.1, XBB.2.3 or the previous XBB variant,^35^ and the BA.2.86 variant can evade nAbs produced in response to the XBB variant that is directed against various epitopes.^36^ Various scenarios suggest that infection with new variants or immunization with updated antigens has reduced protection against future variants. The ongoing COVID-19 pandemic and the consequent continued evolution of SARS-CoV-2 will continue to give rise to additional mutations or recombinant strains. Therefore, a vaccine that can provide relatively long-lasting protection against future emerging variants is needed.

The RBD is the main target of existing SARS-CoV-2 vaccines, which have been shown to have a favorable safety profile. In this study, we propose a novel RBD design strategy. The cRBD sequences were designed by incorporating mutations that promote the immune evasion of different strains into the original RBD sequence, with the aim of preserving the immunogenicity of the original RBD while conferring protective efficacy against different variants. The reference variants for this sequence were mainly the BA.5 variant and previously emerged VOCs. Serum from Balb/c mice immunized with the cRBD vaccines showed neutralizing activity against subsequent VOCs and exhibited protective efficacy against strains such as EG.5.1. This result suggests that vaccines designed according to a similar approach can achieve some coverage against future emerging variants and provides a new idea for the development of a broad-spectrum vaccine against COVID-19. Although traditional polyvalent and multimeric vaccines can also provide protection against multiple mutants,^16,17^ the cRBD-based vaccine was shown to provide protection against newer emerging strains such as EG.5.1.

Vaccine-induced serum-neutralizing activity is directly related to the protective efficacy of the vaccine.^28,37,38^ In the present study, we primarily selected VOCs and evaluated the levels of nAbs against them in postimmunization serum to evaluate the protective efficacy of the vaccine against different strains. Serum from Balb/c mice immunized with cRBD showed some neutralization activity against all tested variants, although the neutralization activity varied among the strains. This finding indicates that the vaccine we designed has broad protective efficacy against different SARS-CoV-2 variants, which was also confirmed by virus challenge experiment. In K18-hACE2 model mice, high levels of specific binding antibodies were detected in serum after immunization, but no neutralizing antibodies were detected. However, the vaccine still exerted a certain protective effect in the challenge test, indicating that a large number of non-neutralizing antibodies were induced by the vaccine in hACE2 mice, and they may play a protective role through antibody-dependent cellular cytotoxicity, antibody-dependent cellular phagocytosis or complement activation.^39^

We designed three cRBD sequences in this study, and most of their mutation sites were basically the same, but there were large differences in the breadth of activity of the induced nAbs. When we take the first sequence as the basis, the difference between cRBD2 and cRBD1 is that the G339D mutation site is eliminated and the S371F mutation site is added to cRBD2, whereas cRBD3 contains three additional mutations, F486V, D405N, and R408S, relative to cRBD2. cRBD and BA.1 RBD did not show significant differences in inducing the production of binding antibodies against BA.2 RBD. cRBD2 induced the narrowest spectrum of nAbs among the three constructs, with relatively low potency against all strains tested. (Fig. 3). However, cRBD2 appeared to regain its protective efficacy after the addition of three mutations, F486V, D405N, and R408S, resulting in cRBD3. cRBD3-induced nAbs showed a similar neutralization spectrum to that of cRBD1. Moreover, cRBD3 produced a slightly lower level of nAbs against the prototype to BA.5 strains than did cRBD1, but it induced higher levels of nAbs against emerging strains such as EG.5.1, EG.5.1.1, XBB.1.5, and XBB.1.16. D405N and R408S are present in the BA.2 variant and all variants that emerged after. It has been reported that these two mutations together with S371F largely account for immune escape.^40^ Therefore, supplementation of constructs with mutations at these sites may account for the broad-spectrum activity of cRBD3-induced antibodies. F486 was mutated in the BA.4/5 variant and all variants after, but this site was mutated to valine only in the BA.4/5, BQ.1.1, and BF.7 variants, consistent with cRBD3, and to proline in the remaining strains assayed. Mutations in F486 are also important for immune evasion,^40^ but the effect of mutating this site to valine or proline on immune evasion has not been reported. Thus, further experiments are needed to determine whether these mutations play important roles independently or together.

As a preliminary exploration, this study has several limitations. First, animal experiments were performed only in mice and were limited by the relatively small number of animals in each group. Second, we did not set up the high and low doses appropriately, so that the two doses did not show a significant difference between them.

A broad-spectrum vaccine is a type of vaccine that is designed to protect against multiple strains or subtypes of a particular virus or bacterium, rather than just a single strain or subtype. This type of vaccine is developed to provide broader protection against a range of different pathogens. It is important to note that while broad-spectrum vaccines have the potential to protect against a range of pathogens, they may not be as effective as vaccines designed specifically for a single strain or subtype.^17,41^ In Balb/c mice, nAbs induced by the cRBD protein have neutralizing activity against a range of variants, and even though the neutralizing activity against some variants is reduced compared with others, it is still higher than that of the BA.1 RBD. Compared with the BA.1 RBD, the cRBD-induced antibodies showed a 5- to 10-fold increase in the level of nAbs against BA.5 and previously emerged strains, and even more than a 10-fold increase in the level of nAbs against some of the newly emerging strains, suggesting a broader spectrum of antibodies, and indirectly, a broad range of protective efficacy. Furthermore, there is still room for optimization, and the possibility that the emergence of new mutation sites in the future will lead to the loss of protective efficacy afforded by the currently optimal construct cannot be excluded. The results confirm the feasibility of adding mutations that promote immune evasion to the original RBD to develop a vaccine with a broad protective effect against SARS-CoV-2. Our research offers new insights into the development of broad-spectrum SARS-CoV-2 vaccines, and we hope to obtain more broad-spectrum and effective vaccines based on this method through further optimization of antigens.

## Materials and methods

### Prediction of B cell epitopes

The prediction and scoring of RBD protein B-cell epitopes was performed by the IEBD online program (https://www.iedb.org/).

### Virus and animals

The SARS-CoV-2 strains used in this study were all from the National Kunming High-level Biosafety Primate Research Center. Determine virus titer through plaque assay. All virus operations were performed in BSL3/4 laboratory, and all operations followed the corresponding regulations of biosafety laboratory.

The animals (BALB/C mice, 6–8 weeks old, female; K-18 hACE2 mice, 6–8 weeks old, female) were all from the Institute of Medical Biology, Chinese Academy of Medical Sciences (Kunming, China) (Manufacturing license: SCXK (DIAN)2022-0002). All animal experiments have been approved by the Committee on the Ethics of Experimental Animals (approval ID: DWSP202306022).

### Protein expression and purification

The gene sequences of the designed cRBD protein and BA.1 RBD protein were codon-optimized for expression in *E.coli*. The protein gene sequences were inserted into the pet-28a (+) plasmid and then transformed into BL21 (DE3), induced for expression with isopropyl-β-D-thiogalactopyranoside (IPTG) at 37 °C for 6 h. Cells were collected by centrifugation, lysed, and the precipitate was collected by centrifugation. The precipitate was resuspended in washing buffer (20 mM Tris-HCl, 0.5 M NaCl, 2 M urea, 1%Triton-X100, pH 8.0) and then collected by centrifugation and repeating the process once. The inclusion body protein was dissolved in inclusion body solubilization buffer (20 mM Tris-HCl, 0.5 M NaCl, 8 M urea, 20 mM β-mercaptoethanol, pH 8.0), the supernatant was collected and purified by Ni affinity chromatography. The eluate at the peak of the elution was slowly added dropwise to refolding buffer (20 mM Tris-HCl, 0.5 mM NaCl, 2 M urea, 55 mM glucose, 2 mM GSH, 0.2 mM GSSG, 20% glycerol, pH 8.0), incubated overnight at 4 °C, and the refolded solution was concentrated using a 10 kDa molecular weight cut-off ultrafiltration membrane. The concentrated solution was dialyzed against citrate buffer (pH 4.0) containing 0.02% EDTA-2Na, and then further concentrated using PEG20000. The recombinant protein was analyzed by 10% SDS-PAGE and Western blot (primary antibodies: HL257; GeneTex; second antibody: RS0002, Immunoway) analysis was performed. Protein quantification was carried out using the Bradford (P0006-1, Beyotime), and experiments were performed according to the instructions of the kit.

### Protein structure and protein-protein interaction prediction

Alphafold3 (https://alphafoldserver.com) was used to predict the RBD and hACE2 structures and their interactions. The Proteinview (v0.1.0) plug-in in VScode (v1.92.1) software was used to visualize the prediction results and to find the interaction sites.

### Surface plasmon resonance analysis

Surface plasmon resonance analysis was performed by Biacore 8 K (GE Healthcare). hACE2-Fc were captured at a concentration of 100 response units on Sensor Chip Protein A. cRBD and BA.1 RBD proteins were run across the chip at a range of concentrations for kinetic analysis, while additional channels were set up as controls. Bound samples were dissociated using HBS-EP+ running buffer running at a flow rate of 30 μl/min for 300 s. The sensor chip was regenerated using regeneration buffer (glycine pH 5.0) for 60 s before the next experiment. The association (Ka) and dissociation (Kd) rate constant were measured and the affinity constant KD was calculated. The lower the KD value, the stronger the binding ability of the protein to hACE2.

### Vaccine preparation and immunization regimen for mice

The purified proteins were diluted to different concentrations with buffer and mixed with adjuvant (vac-as03-10, InvivoGen) in the appropriate volume to obtain a vaccine suitable for immunization. Four protein sequences were administered in doses of 5 μg and 25 μg each, with an additional adjuvant group serving as the control group.

### Binding antibody assay

96-well plate (442404, Thermo Fisher Scientific) were coated with 1 μg/ml BA.2 RBD protein (440592-V08H123, Sino Biological) overnight at 4 °C. After washing with PBS containing 0.05% Tween-20 (PBST) for three times, the plate was blocked with PBST solution containing 2% BSA at 37 °C for 1 h. The serum samples were serially diluted starting at 1:100 with dilution buffer (0.5% BSA in PBST) and 100 μl of each dilution was added to the plate. After an hour of incubation at 37 °C, the plate was washed three times with PBST. HRP-conjugated goat anti-mouse IgG (A-10,668, Invitrogen) was diluted 1:30,000 in sample dilution buffer and 100 μl was added to each well. The plate was then incubated again at 37 °C for 1 h, followed by three washes with PBST. Subsequently, 100 μl of substrate solution (34,028, Thermo Fisher Scientific) was added to the plate and incubated at room temperature for 15 min. The reaction was stopped using a stop solution (C1058, Solarbio). The absorbance at 450 nm and 630 nm was measured. The well without serum samples served as the blank control, with a threshold value set at 2.1 times the control value for a positive result. The titer of RBD-specific IgG was determined as the reciprocal of the highest dilution of serum where OD_450-630nm_ was equal to or greater than the cutoff value (OD_450-630nm_ > 0.1).

### Authentic virus neutralization experiment

Mouse serum was initially diluted at 1:16 followed by two-fold serial dilutions, with a volume of 50 μl per well. The SARS-CoV-2 virus was diluted in virus maintenance medium to a virus titer of 100 TCID_50_/50 μl. The diluted virus solution was added in equal volumes to the serum at different dilutions. The mixture was gently mixed by pipetting and then incubated at 37 °C with 5% CO_2_ for 1 h. Vero cells were digested, counted, and resuspended to a concentration of 100,000 cells/ml. Subsequently, 100 μl of Vero cells (10,000 cells/well) were added to the virus-serum mixture in the 96-well plate, and the plate was incubated at 37 °C with 5% CO_2_. Cytopathic effects (CPE) were observed daily, and the final results were determined on the 5th day. The reciprocal of the dilution of serum capable of protecting 50% of cells from CPE effects was the antibody titer.

### Pseudoviruses neutralization experiment

A total of 5 SARS-CoV-2 pseudoviruses were employed to evaluate the overall neutralizing antibody titers in the serum of immunized mice. Pseudoviruses BQ.1.1, BF.7, and CH.1.1 were obtained from Genomediteh (GM-0220PV105, GM-0220PV108, GM-0220PV100). Pseudoviruses JN.1 and BA.2.86 were detected with assistance from the research group of Youchun Wang. In brief, the serum to be tested was first heat-inactivated at 56 °C for 30 min. Starting with an initial dilution of 1:20, the serum was serially diluted threefold in a 96-well plate, with a final volume of 100 μl per well of culture medium. The pseudovirus titer was diluted to 2 × 10^6^ TCID_50_/ml using a complete culture medium, with 50 μl added to each well after mixing with the serum and incubating at 37 °C for 1 h. 100 μl of culture medium was also mixed with pseudovirus as a positive control in each well. HACE2-293T cells were digested and suspended at a live cell density of 5 × 10^5^/ml, and 100 μl was added to each well, followed by incubation in a 5% CO_2_ incubator for 48 h. After removing the culture medium, 100 μl of DPBS was added to each well, and 100 μl of luciferase assay reagent (6066769, PerkinElmer) was added. The relative light units were read after three minutes to represent luciferase activity. The neutralizing antibodies were determined as the reciprocal of the serum dilution that inhibited 50% of luciferase activity, representing neutralization of 50% of viral infection, calculated using GraphPad.

### Histopathology

The tissues were fixed in 10% formalin for 3–7 days. After embedding in paraffin, the tissues were sectioned into 5 μm slices for hematoxylin and eosin (HE) staining. The sections were scanned by 3DHISTECH. The HE-stained sections were evaluated by experienced pathologists using the CaseViewer provided by the manufacturer for scoring. The scoring criteria include inflammatory cell infiltration, lung hemorrhage, vascular thrombosis, obstruction and protein exudation in bronchial, and lung septal thickening and consolidation. The scores for each criterion were summed to obtain a total lung pathology score, reflecting the severity of lung pathology in the mice.

### ELISPOTS assay

The assay was performed following the manufacturer’s instructions (Mabtech). Briefly, the spleens were harvested post-mortem, and single-cell suspensions were obtained by mechanical disruption on a cell strainer. Lymphocytes were then isolated following the protocol provided in the kit (P8860, Solarbio). The ELISPOT plates (3321-4APT-10, 3441-4APW-10, 3311-4APW-2, Mabtech) were pre-coated and equilibrated as instructed, and the isolated lymphocytes were enumerated and seeded into the plates, followed by the addition of the stimulant (2 μg/well). Specific procedure was carried out according to the kit instructions.

### Detection of inflammatory factors in serum

The Mouse Cytokine 23-Plex immunoassay using the Bio-Plex Pro kit was carried out on the Bio-Plex system (Bio-Rad) following the manufacturer’s protocol. The inflammatory cytokines analyzed included Eotaxin, G-CSF, GM-CSF, IFN-g, IL-1a, IL-1b, IL-2, IL-3, IL-4, IL-5, IL-6, IL-9, IL-10, IL-12, IL-12 (p70), IL-13, IL-17A, KC, MCP-1, MIP-1a, MIP-1b, RANTES, and TNF-α. Cytokines that were below the detection limit or considered unreliable by the instrument were omitted from the text.

### mRNA sequencing

Following the third immunization on day 63D, whole blood samples were collected from mice for sequencing. Total RNAs were extracted from the blood using Trizol Reagent (cat. NO 15596026, Invitrogen). 2 μg total RNAs were used for stranded RNA sequencing library preparation using KC-DigitalTM Stranded mRNA Library Prep Kit for Illumina® (Catalog NO. DR08502, Wuhan Seqhealth Co., Ltd. China) following the manufacturer’s instruction. The library products corresponding to 200-500 bps were enriched, quantified, and finally sequenced on DNBSEQ-T7 sequencer (MGI Tech Co., Ltd. China) with PE150 model.

### BCR sequencing

RNA extraction testing was performed in the same way as for mRNA sequencing. About 2 μg total RNA of each sample was used for BCR sequencing library preparation using KC-DigitalTM Stranded BCR-seq Library Prep Kit for Illumina® 150 (Seqhealth Technology Co., Ltd., Wuhan, China, Cat. No. DT0815-02) following the manufacturer’s instruction. The library products corresponding to 250-500 bp were enriched, quantified, and finally sequenced on NovaSeq (Illumina®). The sequence was mapped to the international ImMunoGeneTics (IMGT) database. And using MiXCR software (version 3.0.3) to obtain V, D, and J fragments, rearrangement, and CDR3 sequences.

### Animals attacked by virus

The mice that completed the whole immune process were transferred to ABSL3 Laboratory for animal virus tests. The virus attack test will be conducted after the immunization of the experimental animals, mice were given 100 μl (1 × 10^6^ TCID_50_) of virus solution. In short, the mice were anesthetized with isoflurane (RWD R510-22-4) Inhalational anesthetic, and then the virus was dripped into the nasal cavity of the mice several times, and the mice were put back into the cage after the attack.

### Sampling and monitoring of animals

Laboratory animals were sampled under isoflurane anesthesia using a mouse-specific sampling swab. When taking a nasal swab, use a sampling swab to gently scrape the nasal cavity and nasal secretions into a centrifuge tube containing 800 μl Trizol. When taking a throat swab, the mouse’s oral cavity was gently opened and swabbed for throat and pharyngeal secretions, which were placed into a centrifuge tube containing 800 μl Trizol. The body temperature and weight of the mice were measured under anesthesia.

### Animal dissection

Mice anesthetized with isoflurane were killed by cervical dislocation, and then dissected by professional operators. The lungs, turbinate, and organs of the mice were obtained and weighed. The obtained tissues were divided into two parts, one was fixed in 4% paraformaldehyde for pathological detection and the other was added in 800 μl Trizol for viral load determination.

### Viral load determination

The tissue was homogenized before viral RNA was extracted. RNA extraction was performed using the Kingfisher Flex Purification System (ThermoFisher) automated nucleic acid extractor and the MagMAX-96 nucleic acid extraction kit (ThermoFisAM18361836). Operate according to manufacturer’s instructions. Taqman Fast Viral1-Step Master Mix (ThermoFisher, 4444432) was used for qPCR. The N gene was used to determine the viral genome copy number; the E gene was used to determine the subgenomic copy number. First, 2.5 μl PCR MIX with 0.5 μl upstream and downstream primers was added (gRNA-N-F: GACC-CCAAAATCAGCGAAAT; gRNA-N-R: TCTGGTTACT-GCCAGTTGAATCTG) (sgRNA-E-F: CGATCTCTTGTA-GATCTGTTCTC; sgRNA-E-R: ATATTGCAGCAGTACG-CACACA), 0.5 μl Probe (gRNA-N: FAM-ACCCC-GCATTACGTTTGGTGGACC-BHQ1) (sgRNA-E-FAM: ACACTAGCCATCCTTACTGCGCTTCG-BHQ1), 3.5 μl H_2_O and 2.5 μl RNA templates into 384-well plates. The reaction was then performed on a CFX384 fluorescent quantitative PCR (Bio-Rad). After the reaction was completed, the data were collected and the viral genome or subgenome copy number was calculated according to the standard.

### Statistical analysis

The data were statistically analyzed and plotted using Excel, GraphPad Prism 8.0.2, and Origin 2023. Values were presented as mean ± SD. One-way analysis of variance (ANOVA) combined with Tukey’s post hoc test was used to determine most statistical significance between groups. Statistical analysis of differences in IGH expression levels among the groups was conducted using multiple tests and corrected for FDR, with Scheffe performing post hoc tests.

## Supplementary information

Supplementary information

## Data Availability

All study data are included in the article and/or supplementary information. The original data of mRNA and BCR sequencing in this study is available at GEO under the accession GSE261311 and GSE261313.

## References

[1] *WHO COVID-19 Dashboard*. https://covid19.who.int/ (2023).

[2] Wang, M.-Y. et al. SARS-CoV-2: structure, biology, and structure-based therapeutics development. *Front Cell Infect. Microbiol.* **10**, 587269 (2020). 33324574 10.3389/fcimb.2020.587269 PMC7723891

[3] Magazine, N. et al. Mutations and evolution of the SARS-CoV-2 spike protein. *Viruses* **14**, 640 (2022). 35337047 10.3390/v14030640 PMC8949778

[4] Raghuvamsi, P. V. et al. SARS-CoV-2 S protein: ACE2 interaction reveals novel allosteric targets. *eLife* **10**, 63646 (2021). 10.7554/eLife.63646 PMC7932696 33554856

[5] Dai, L. & Gao, G. F. Viral targets for vaccines against COVID-19. *Nat. Rev. Immunol.* **21**, 73–82 (2021). 33340022 10.1038/s41577-020-00480-0 PMC7747004

[6] Hu, B., Guo, H., Zhou, P. & Shi, Z. L. Characteristics of SARS-CoV-2 and COVID-19. *Nat. Rev. Microbiol.* **19**, 141–154 (2021). 33024307 10.1038/s41579-020-00459-7 PMC7537588

[7] Hattab, D., Amer, M. F. A., Al-Alami, Z. M. & Bakhtiar, A. SARS-CoV-2 journey: from alpha variant to omicron and its sub-variants. *Infection* **52**, 767–786 (2024). 38554253 10.1007/s15010-024-02223-y PMC11143066

[8] Garcia-Beltran, W. F. et al. Multiple SARS-CoV-2 variants escape neutralization by vaccine-induced humoral immunity. *Cell* **184**, 2523 (2021). 33930298 10.1016/j.cell.2021.04.006 PMC8082941

[9] Hoffmann, M. et al. The Omicron variant is highly resistant against antibody-mediated neutralization: implications for control of the COVID-19 pandemic. *Cell* **185**, 447–456.e411 (2022). 35026151 10.1016/j.cell.2021.12.032 PMC8702401

[10] Andrews, N. et al. Covid-19 vaccine effectiveness against the Omicron (B.1.1.529) variant. *N. Engl. J. Med.* **386**, 1532–1546 (2022). 35249272 10.1056/NEJMoa2119451 PMC8908811

[11] Zhao, F., Zai, X., Zhang, Z., Xu, J. & Chen, W. Challenges and developments in universal vaccine design against SARS-CoV-2 variants. *NPJ Vaccines* **7**, 167 (2022). 36535982 10.1038/s41541-022-00597-4 PMC9761649

[12] Planas, D. et al. Considerable escape of SARS-CoV-2 Omicron to antibody neutralization. *Nature* **602**, 671–675 (2022). 35016199 10.1038/s41586-021-04389-z

[13] Liu, L. et al. Striking antibody evasion manifested by the Omicron variant of SARS-CoV-2. *Nature* **602**, 676–681 (2022). 35016198 10.1038/s41586-021-04388-0

[14] Wang, Q. et al. Alarming antibody evasion properties of rising SARS-CoV-2 BQ and XBB subvariants. *Cell* **186**, 279–286.e278 (2023). 36580913 10.1016/j.cell.2022.12.018 PMC9747694

[15] Datta, A., Kapre, K., Andi-Lolo, I. & Kapre, S. Multi-valent pneumococcal conjugate vaccine for global health: from problem to platform to production. *Hum. Vaccin. Immunother.* **18**, 2117949 (2022). 36239596 10.1080/21645515.2022.2117949 PMC9721430

[16] Wang, R. et al. Development of a thermostable SARS-CoV-2 variant-based bivalent protein vaccine with cross-neutralizing potency against Omicron subvariants. *Virology* **576**, 61–68 (2022). 36174448 10.1016/j.virol.2022.09.003 PMC9486464

[17] Liang, Y. et al. Design of a mutation-integrated trimeric RBD with broad protection against SARS-CoV-2. *Cell Discov.* **8**, 17 (2022). 35169113 10.1038/s41421-022-00383-5 PMC8847466

[18] Vita, R. et al. The Immune Epitope Database (IEDB): 2018 update. *Nucleic Acids Res.* **47**, D339–D343 (2019). 30357391 10.1093/nar/gky1006 PMC6324067

[19] Greaney, A. J. et al. Comprehensive mapping of mutations in the SARS-CoV-2 receptor-binding domain that affect recognition by polyclonal human plasma antibodies. *Cell Host Microbe* **29**, 463–476.e466 (2021). 33592168 10.1016/j.chom.2021.02.003 PMC7869748

[20] Carabelli, A. M. et al. SARS-CoV-2 variant biology: immune escape, transmission and fitness. *Nat. Rev. Microbiol* **21**, 162–177 (2023). 36653446 10.1038/s41579-022-00841-7 PMC9847462

[21] Starr, T. N. et al. Prospective mapping of viral mutations that escape antibodies used to treat COVID-19. *Science* **371**, 850–854 (2021). 33495308 10.1126/science.abf9302 PMC7963219

[22] Weisblum, Y. et al. Escape from neutralizing antibodies by SARS-CoV-2 spike protein variants. *Elife* **9**, 61312 (2020). 10.7554/eLife.61312 PMC7723407 33112236

[23] Piccoli, L. et al. Mapping neutralizing and immunodominant sites on the SARS-CoV-2 spike receptor-binding domain by structure-guided high-resolution serology. *Cell* **183**, 1024–1042.e1021 (2020). 32991844 10.1016/j.cell.2020.09.037 PMC7494283

[24] Ke, Q. et al. Non-glycosylated SARS-CoV-2 RBD elicited a robust neutralizing antibody response in mice. *J. Immunol. Methods* **506**, 113279 (2022). 35533747 10.1016/j.jim.2022.113279 PMC9075978

[25] Takatsu, K., Kouro, T. & Nagai, Y. Interleukin 5 in the link between the innate and acquired immune response. *Adv. Immunol.* **101**, 191–236 (2009). 19231596 10.1016/S0065-2776(08)01006-7

[26] Vazquez, M. I., Catalan-Dibene, J. & Zlotnik, A. B cells responses and cytokine production are regulated by their immune microenvironment. *Cytokine* **74**, 318–326 (2015). 25742773 10.1016/j.cyto.2015.02.007 PMC4475485

[27] He, B. et al. Comparative global B cell receptor repertoire difference induced by SARS-CoV-2 infection or vaccination via single-cell V(D)J sequencing. *Emerg. Microbes Infect.* **11**, 2007–2020 (2022). 35899581 10.1080/22221751.2022.2105261 PMC9377262

[28] Khoury, D. S. et al. Neutralizing antibody levels are highly predictive of immune protection from symptomatic SARS-CoV-2 infection. *Nat. Med.* **27**, 1205–1211 (2021). 34002089 10.1038/s41591-021-01377-8

[29] Yinda, C. K. et al. K18-hACE2 mice develop respiratory disease resembling severe COVID-19. *PLoS Pathog.* **17**, e1009195 (2021). 33465158 10.1371/journal.ppat.1009195 PMC7875348

[30] Milacic, M. et al. The reactome pathway knowledgebase 2024. *Nucleic Acids Res* **52**, D672–d678 (2024). 37941124 10.1093/nar/gkad1025 PMC10767911

[31] Cao, Y. et al. Omicron escapes the majority of existing SARS-CoV-2 neutralizing antibodies. *Nature* **602**, 657–663 (2022). 35016194 10.1038/s41586-021-04385-3 PMC8866119

[32] Ai, J. et al. Antibody evasion of SARS-CoV-2 Omicron BA.1, BA.1.1, BA.2, and BA.3 sub-lineages. *Cell Host Microbe* **30**, 1077–1083.e1074 (2022). 35594867 10.1016/j.chom.2022.05.001 PMC9080084

[33] Iketani, S. et al. Antibody evasion properties of SARS-CoV-2 Omicron sublineages. *Nature* **604**, 553–556 (2022). 35240676 10.1038/s41586-022-04594-4 PMC9021018

[34] Dejnirattisai, W. et al. SARS-CoV-2 Omicron-B.1.1.529 leads to widespread escape from neutralizing antibody responses. *Cell* **185**, 467–484.e415 (2022). 35081335 10.1016/j.cell.2021.12.046 PMC8723827

[35] Faraone, J. N. et al. Immune evasion and membrane fusion of SARS-CoV-2 XBB subvariants EG.5.1 and XBB.2.3. *Emerg. Microbes Infect.* **12**, 2270069 (2023). 37819267 10.1080/22221751.2023.2270069 PMC10606793

[36] Yang, S. et al. Antigenicity and infectivity characterisation of SARS-CoV-2 BA.2.86. *Lancet Infect. Dis.* **23**, e457–e459 (2023). 37738994 10.1016/S1473-3099(23)00573-X

[37] Gilbert, P. B. et al. Immune correlates analysis of the mRNA-1273 COVID-19 vaccine efficacy clinical trial. *Science* **375**, 43–50 (2022). 34812653 10.1126/science.abm3425 PMC9017870

[38] Bergwerk, M. et al. Covid-19 breakthrough infections in vaccinated health care workers. *N. Engl. J. Med.* **385**, 1474–1484 (2021). 34320281 10.1056/NEJMoa2109072 PMC8362591

[39] Chandler, T. L., Yang, A., Otero, C. E., Permar, S. R. & Caddy, S. L. Protective mechanisms of nonneutralizing antiviral antibodies. *PLoS pathog.* **19**, e1011670 (2023). 37796829 10.1371/journal.ppat.1011670 PMC10553219

[40] Cao, Y. et al. BA.2.12.1, BA.4 and BA.5 escape antibodies elicited by Omicron infection. *Nature* **608**, 593–602 (2022). 35714668 10.1038/s41586-022-04980-y PMC9385493

[41] Zhao, Y. et al. Vaccination with S, an antigen guided by SARS-CoV-2 S protein evolution, protects against challenge with viral variants in mice. *Sci. Transl. Med.* **15**, eabo3332 (2023). 36599007 10.1126/scitranslmed.abo3332

