# Supplementary material for: A protein vaccine of RBD integrated with immune evasion mutation shows broad protection against SARS-CoV-2
Source: Signal Transduct Target Ther. 2024 Nov 6;9:301. doi: 10.1038/s41392-024-02007-8 (PMC11538548; doi:10.1038/s41392-024-02007-8)
Supplement: Supplementary file 1 — Supplementary information [file 41392_2024_2007_MOESM1_ESM.docx]

Supplementary Materials for

A protein vaccine of RBD integrated with immune evasion mutation shows broad protection against SARS-CoV-2

Ran An^1#^, Hao Yang^1#^, Cong Tang^1#^, Qianqian Li^1,2,3#^, Qing Huang^1#^, Haixuan Wang^1^, Junbin Wang^1^, Yanan Zhou^1^, Yun Yang^1^, Hongyu Chen^1^, Wenhai Yu^1^, Bai Li^1^, Daoju Wu^1^, Yong Zhang^1^, Fangyu Luo^1^, Wenqi Quan^1^, Jingwen Xu^1^, Dongdong Lin^1^, Xiaoming Liang^1^, Yuhuan Yan^1^, Longhai Yuan^1^, Xuena Du^1^, Yuxia Yuan^1^, Yanwen Li^1^, Qiangming Sun^1,2,3,4^*, Youchun Wang^1,2,3^*, Shuaiyao Lu^1,2,3,4^*

Correspondence to: Qiangming Sun, qsun@imbcams.com.cn

Youchun Wang, wangyc@nifdc.org.cn

Shuaiyao Lu, lushuaiyao-km@163.com

**This PDF file includes:**

Figures. S1 to S14

**Supplementary information, Fig. S1**


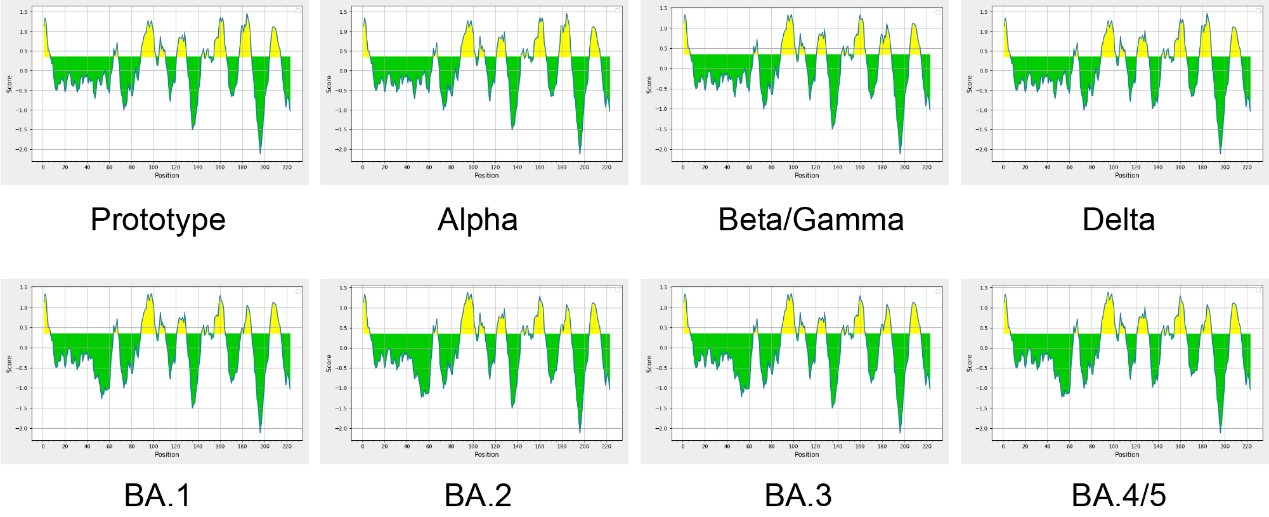


**Supplementary information, Fig. S1** Graph of B cell linear antigenic epitopes prediction of variants.

**Supplementary information, Fig. S2**

**
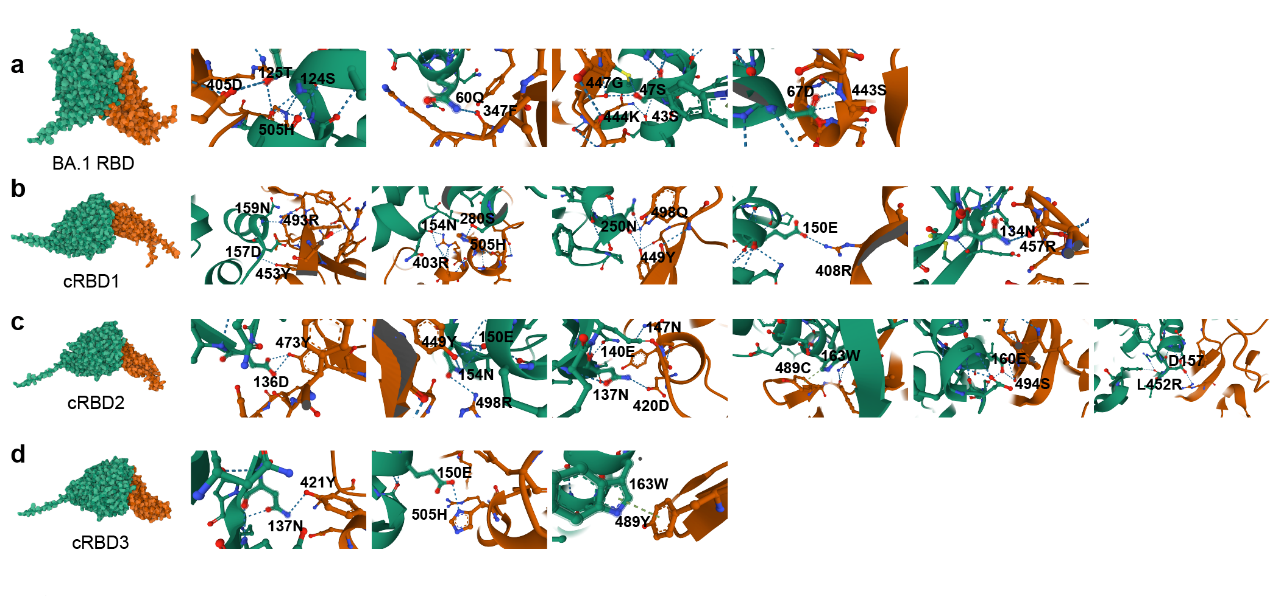
**

**Supplementary information, Fig S2** **The visualization of interaction of hACE2 with BA.1 RBD (a) and cRBD1-3 (b-d).** Specific binding sites are labeled after partial magnification. hACE2 is displayed in green, and cRBD or BA.1 RBD in orange.

**Supplementary information, Fig. S3**


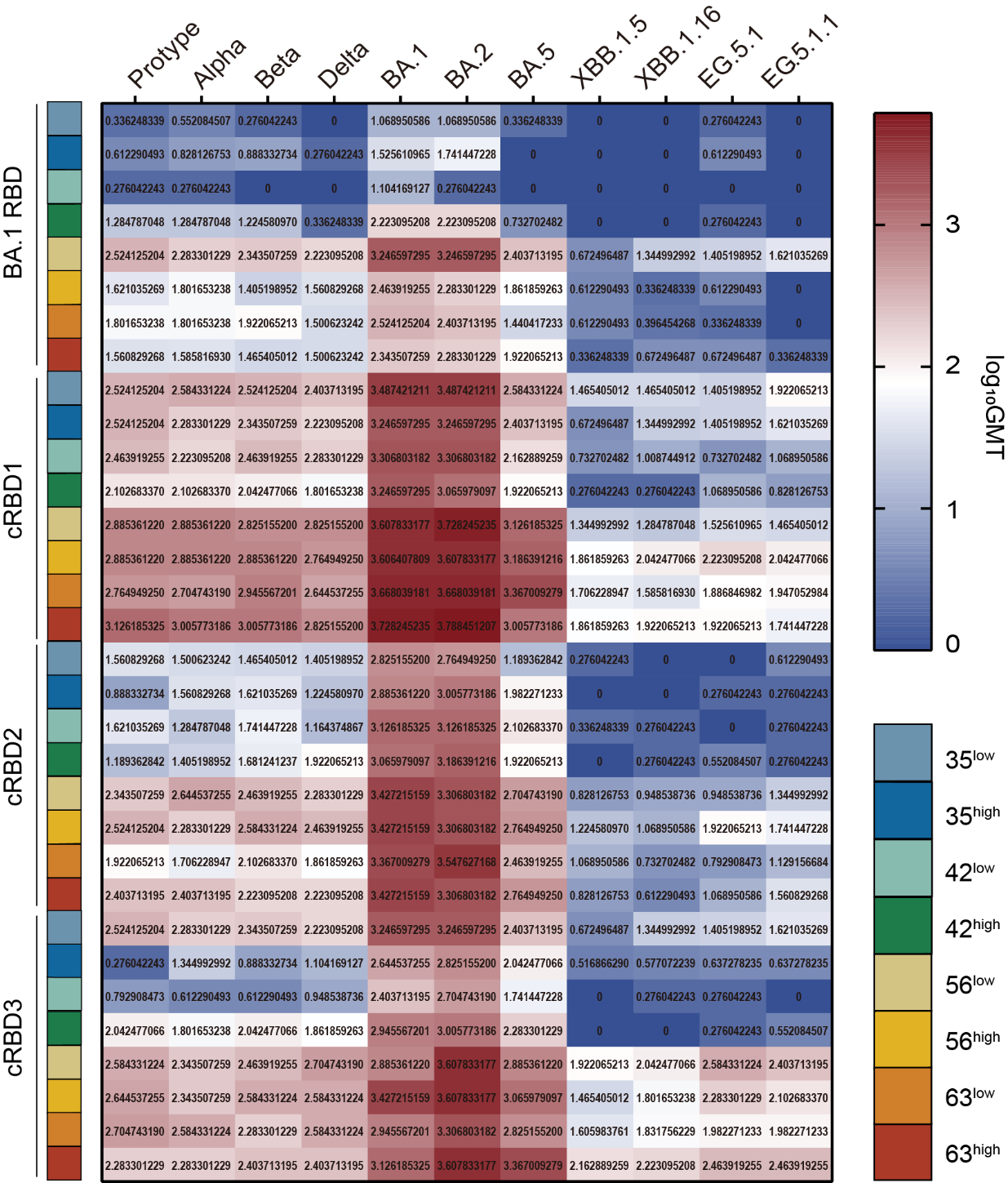


**Supplementary information, Fig. S3 Heatmap of neutralizing antibody results for authentic virus at various time points after immunization of each group (n=5).** The numbers in the color blocks are the log_10_GMT (geometric mean titer). The bluer the color of the color block, the lower the level of neutralizing antibodies, and the redder the color, the higher the level of neutralizing antibodies.

**Supplementary information, Fig. S4**


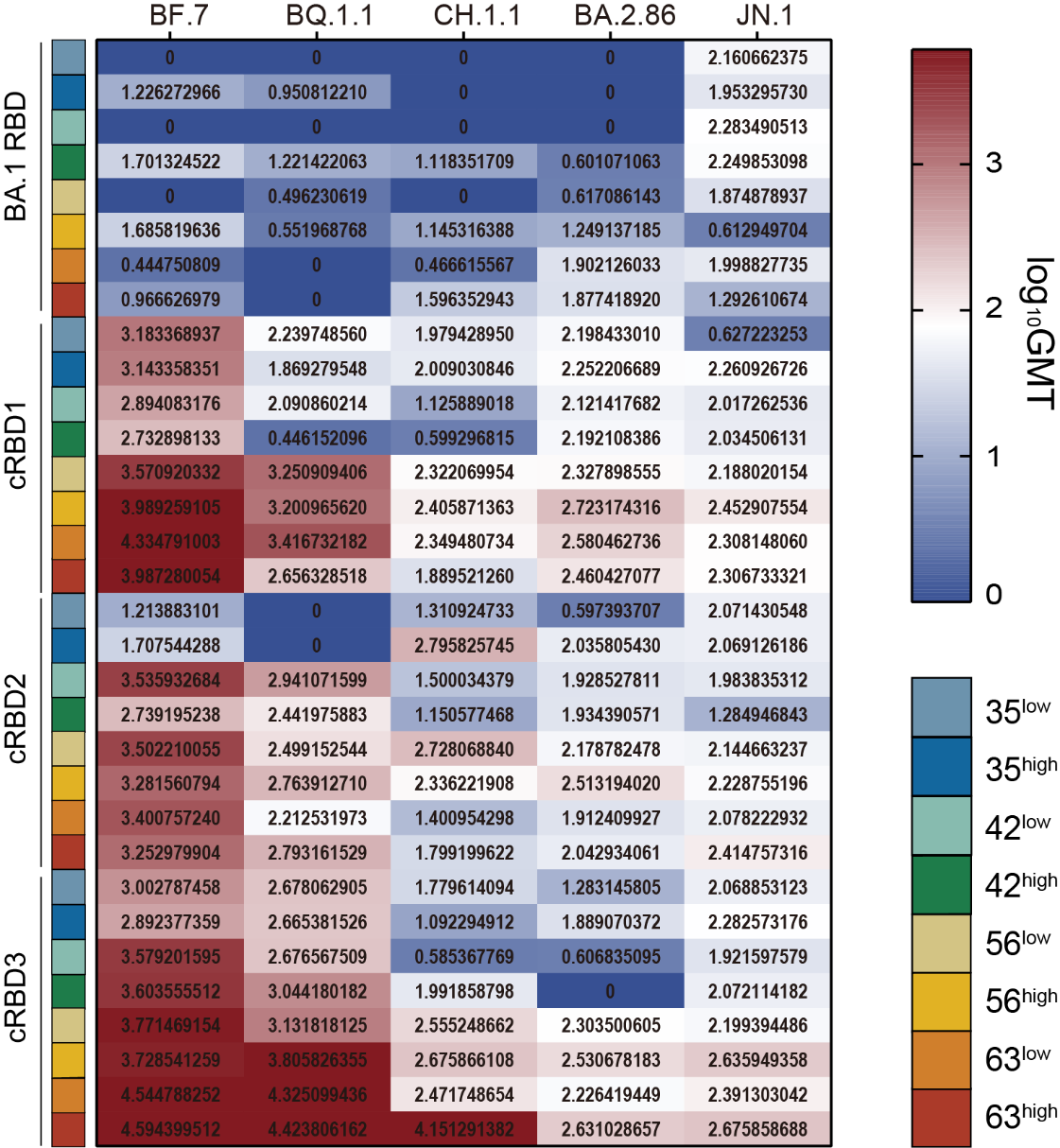


**Supplementary information, Fig. S4 Heatmap of neutralizing antibody results for pseudovirus at various time points after immunization of each group (n=3).** The numbers in the color blocks are the log_10_GMT. The bluer the color of the color block, the lower the level of neutralizing antibodies, and the redder the color, the higher the level of neutralizing antibodies.

**Supplementary information, Fig. S5**

**
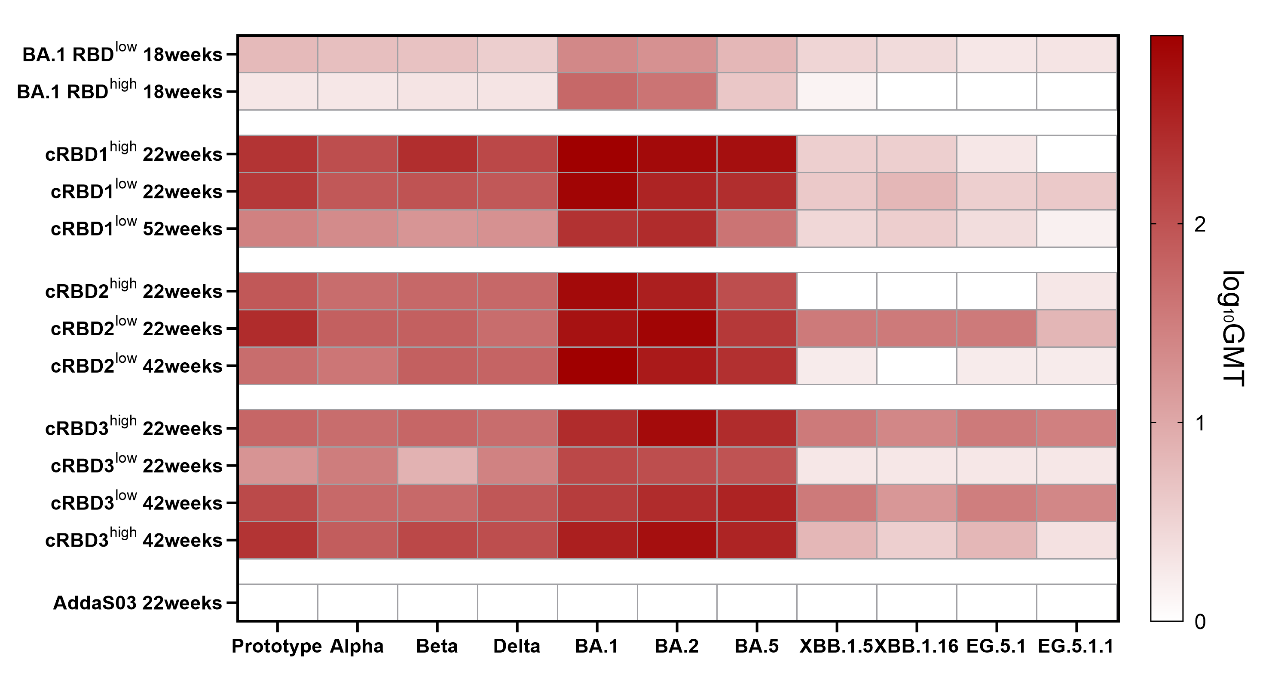
**

**Supplementary information, Fig. S5 Heatmap results of long-term neutralizing antibody in Balb/c mice (n=2-10)**. The redder the color of the color block, the higher the level of neutralizing antibodies.

**Supplementary information, Fig. S6**


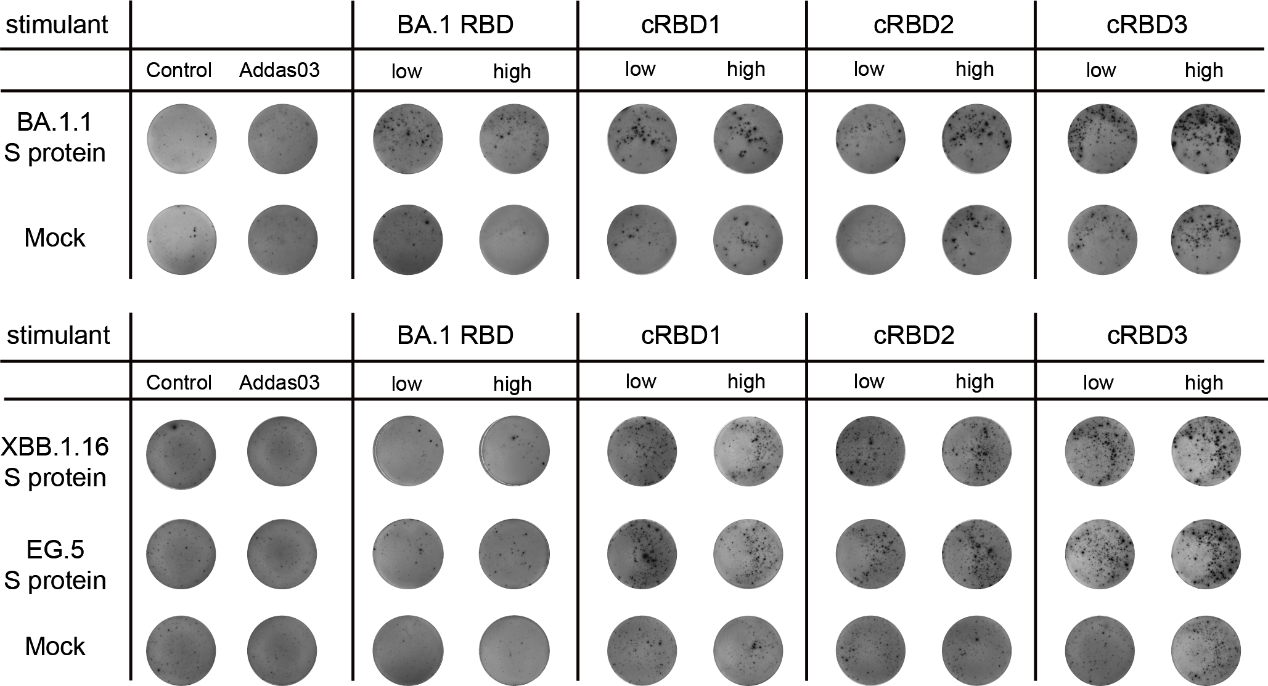


**Supplementary information, Fig. S6 Representative images of ELISPOTS for IFN-γ.** Results using BA.1.1 S protein as a stimulants and results using XBB.1.16, EG.5 as stimulants were separated because the two were not measured at the same time and different mock wells were used.

**Supplementary information, Fig. S7**


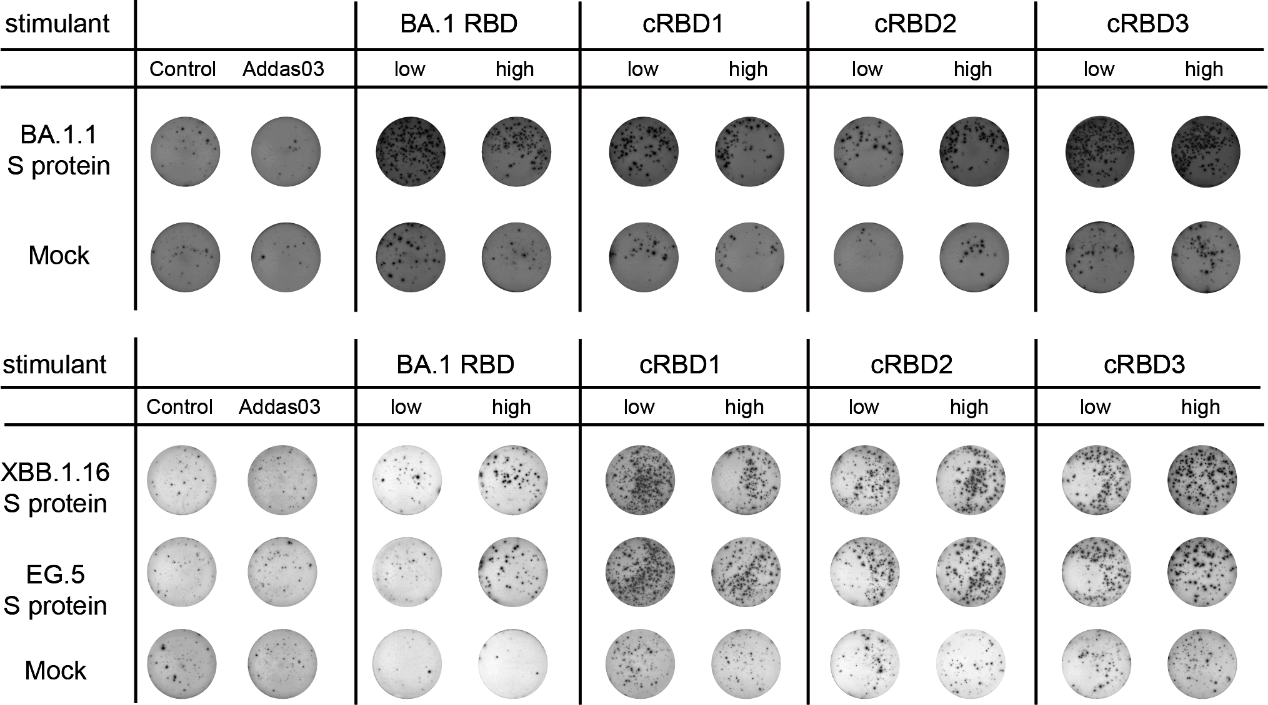


**Supplementary information, Fig. S7 Representative images of ELISPOTS for IL-2.** Results using BA.1.1 S protein as a stimulants and results using XBB.1.16, EG.5 as stimulants were separated because the two were not measured at the same time and different mock wells were used.

**Supplementary information, Fig. S8**


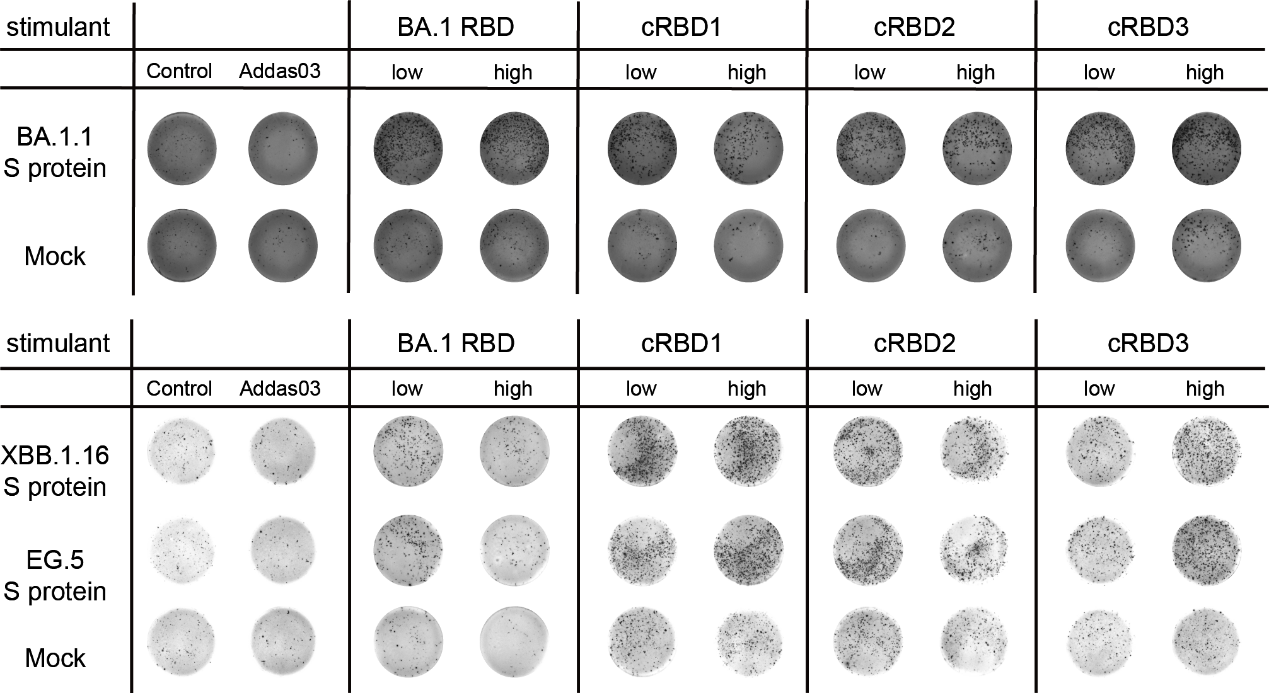


**Supplementary information, Fig. S8 Representative images of ELISPOTS for IL-4.** Results using BA.1.1 S protein as a stimulants and results using XBB.1.16, EG.5 as stimulants were separated because the two were not measured at the same time and different mock wells were used.

**Supplementary information, Fig. S9**


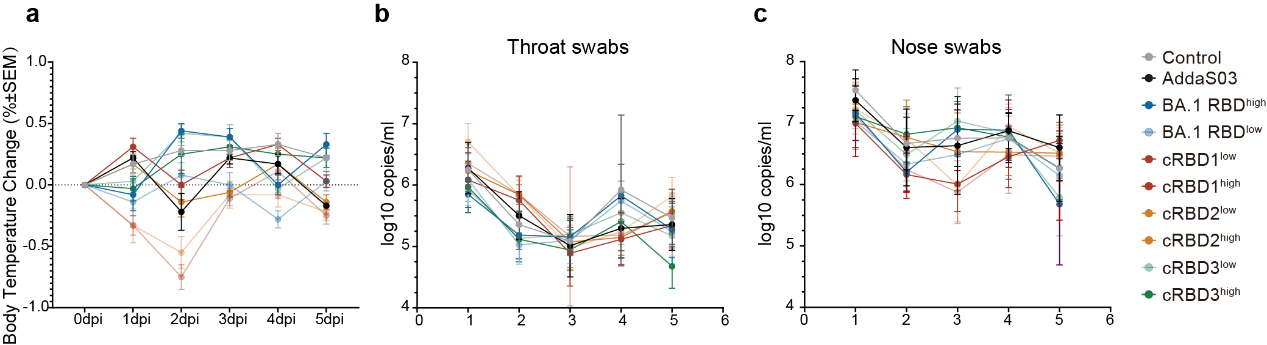


**Supplementary information, Fig. S9 Results of EG.5 challenge in mice after immunization.** **a** Body temperature change after infection in each group(n=10). **b** Throat swab viral load after infection in each group (n=10). **c** Nasal swab viral load after infection in each group (n=10). Data were presented as mean±SD.

**Supplementary information, Fig. S10**


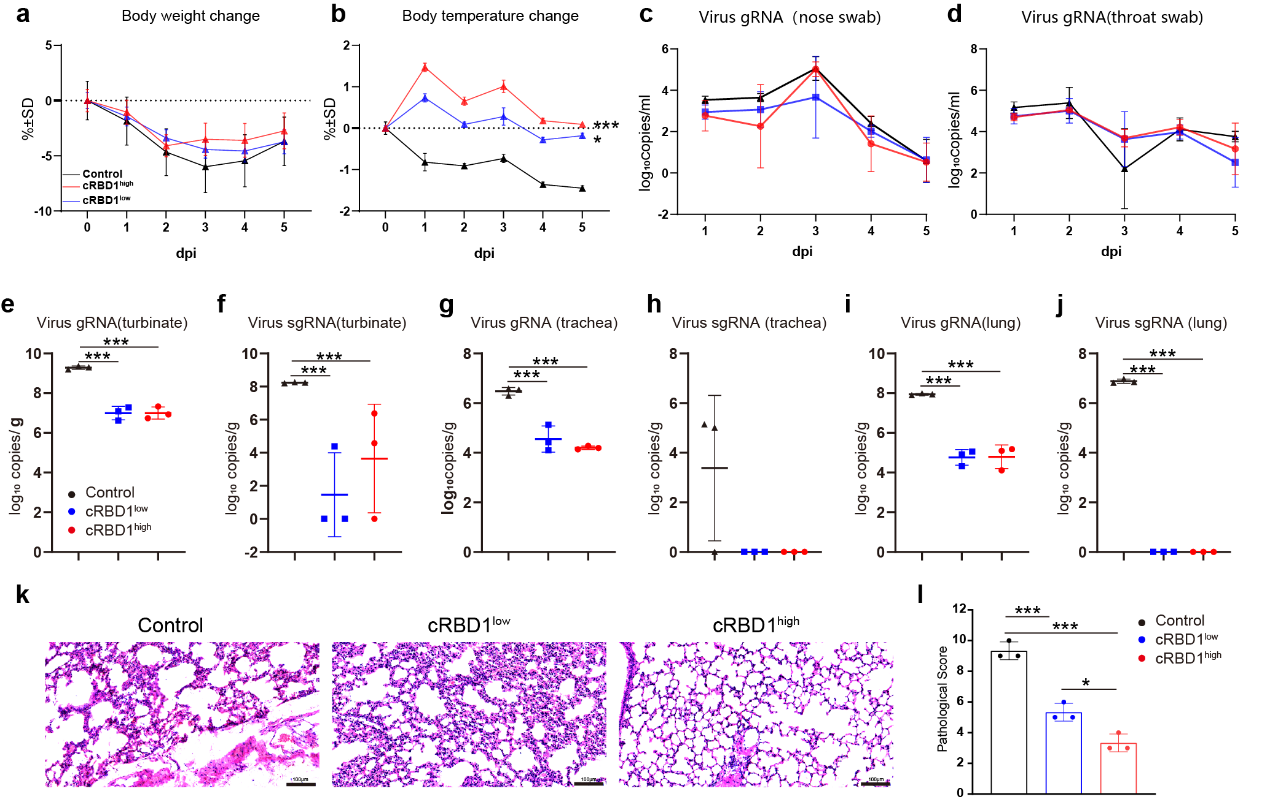


**Supplementary information, Fig. S10 Results of BA.5 challenge in mice after immunized with cRBD1.** n=3 in each group. **a-b** Body weight (a) and body temperature (b) change after infection in each group. **c** Throat swab viral load. **d** Nasal swab viral load. **e-f** Viral genomic (**e**)and subgenomic(**f**) viral loads detected in the turbinate of mice on 5dpi. **g-h** Viral genomic (**g**)and subgenomic(**h**) viral loads detected in the trachea of mice on 5dpi. **i-j** Viral genomic (**i**)and subgenomic(**j**) viral loads detected in the lungs of mice on 5dpi. **k** Histopathological examination of lung. Scale bar, 100μm. **l** Histogram of lung pathology scores. Data are presented as mean ± SD. Results of each group were compared with the control group. Statistical analysis was conducted using one-way ANOVAS and Turkey multiple comparison test for bar graphs. *P < 0.05, **P < 0.01, ***P < 0.005. ns, not significant.

**Supplementary information, Fig. S11**


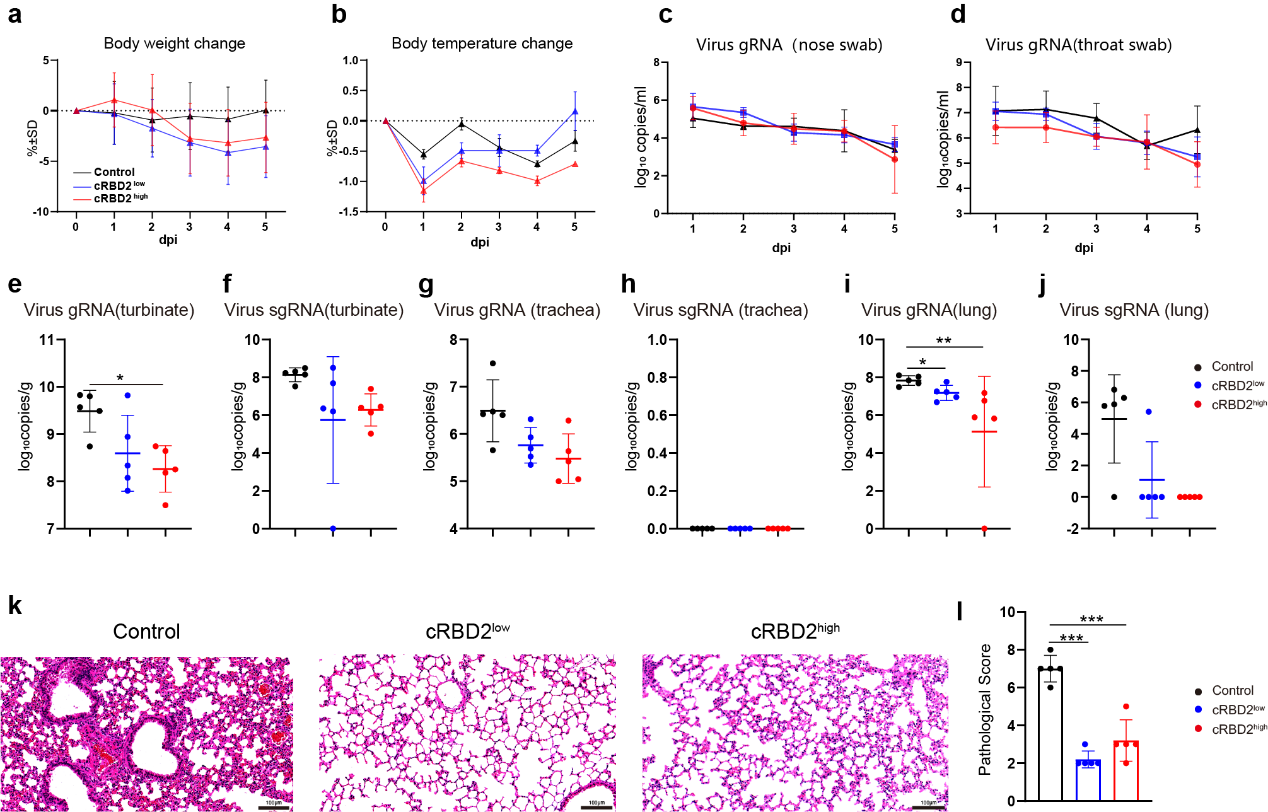


**Supplementary information, Fig. S11 Results of XBB.1.5 challenge in mice after immunized with cRBD2.** n=5 in each group. **a-b** Body weight (a) and body temperature (b) change after infection in each group. **c** Throat swab viral load. **d** Nasal swab viral load. **e-f** Viral genomic (**e**)and subgenomic(**f**) viral loads detected in the turbinate of mice on 5dpi. **g-h** Viral genomic (**g**)and subgenomic(**h**) viral loads detected in the trachea of mice on 5dpi. **i-j** Viral genomic(**i**) and subgenomic(**j**) viral loads detected in the lungs of mice on 5dpi. **k** Histopathological examination of lung. Scale bar, 100μm. **l** Histogram of lung pathology scores. Data are presented as mean ± SEM. Results of each group were compared with the control group. Statistical analysis was conducted using one-way ANOVAS and Turkey multiple comparison test for bar graphs. *P < 0.05, **P < 0.01, ***P < 0.005. ns, not significant.

**Supplementary information, Fig. S12**


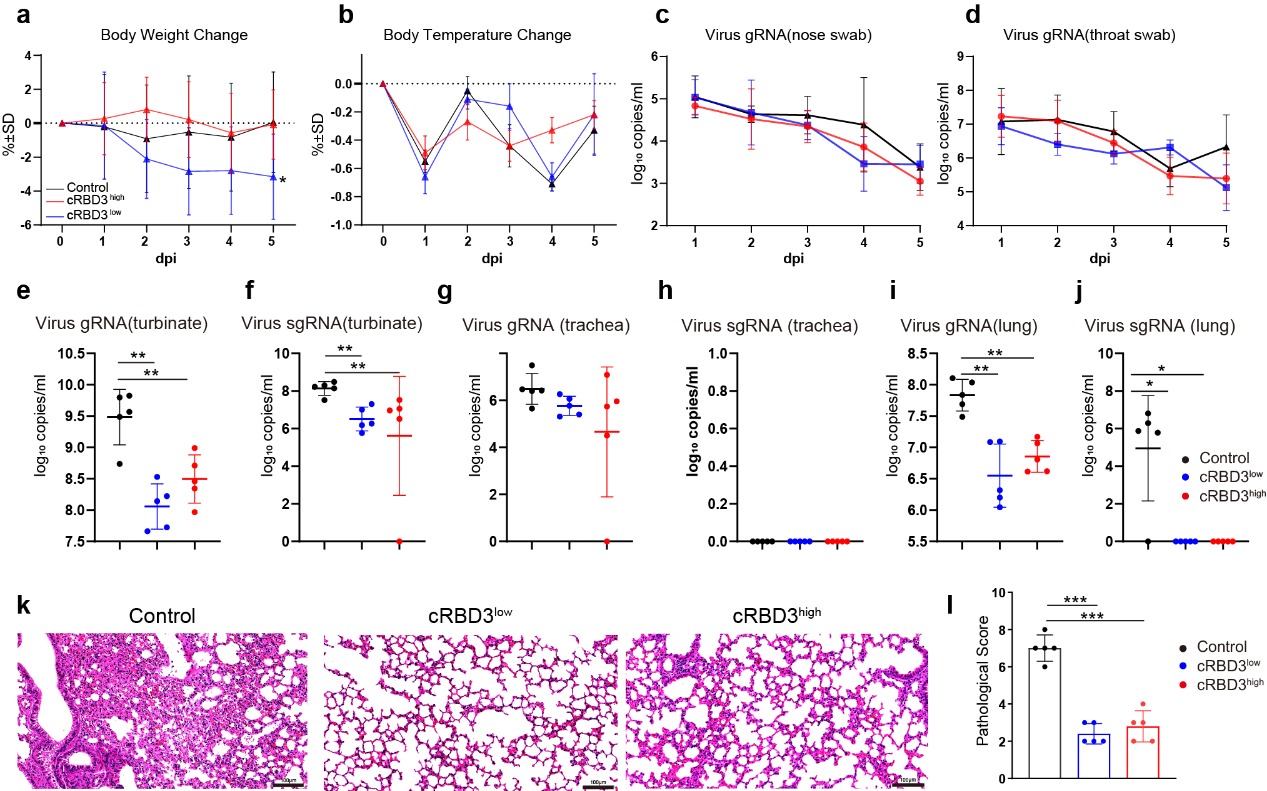


**Supplementary information, Fig. S12 Results of XBB.1.5 challenge in mice after immunized with cRBD3**. n=5 in each group. **a-b** Body weight and body temperature change after infection in each group. **c** Throat swab viral load. d Nasal swab viral load. **e-f** Viral genomic (**e**)and subgenomic(**f**) viral loads detected in the turbinate on 5dpi. **g-h** Viral genomic (**g**)and subgenomic(**h**) viral loads detected in the trachea on 5dpi. **i-j** Viral genomic(**i**) and subgenomic(**j**) viral loads detected in the lungs on 5dpi. **k** Histopathological examination of lung. Scale bar, 100μm. **l** Histogram of lung pathology score. Data are presented as mean±SD. Results of each group were compared with the control group. Statistical analysis was conducted using one-way ANOVAS and Turkey multiple comparison test for bar graphs. *P < 0.05, **P < 0.01, ***P < 0.005. ns, not significant.

**Supplementary information, Fig. S13**

**
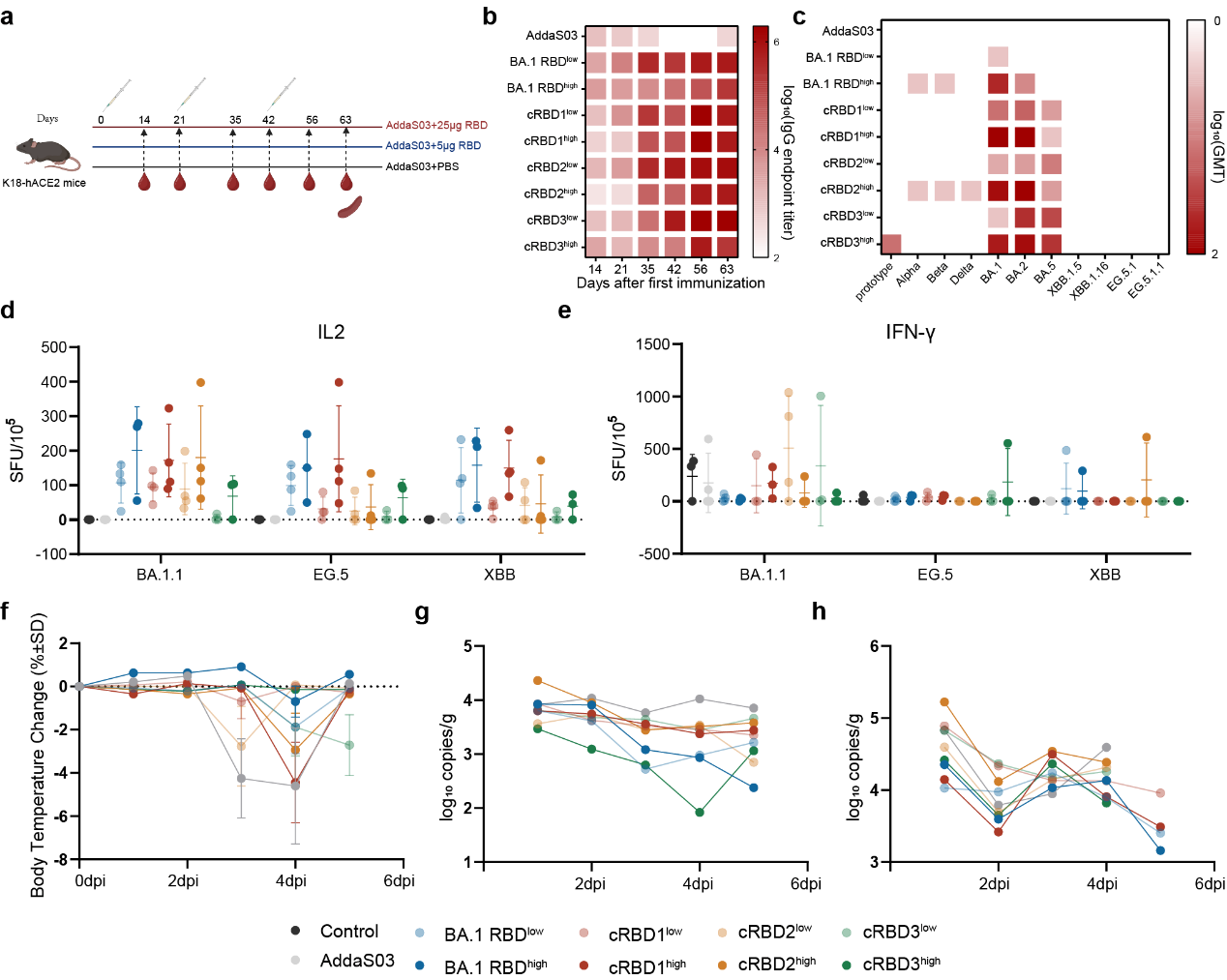
**

**Supplementary information, Fig. S13 Results of immunization and EG.5.1 challenge in K18-hACE2 mice**. n=3 in each group. **a** The timeline of vaccine immunization and sampling (created with BioRender.com). **b** BA.2 RBD-specific binding antibody levels at each time point after immunization in each group. **c** GMT of serum nAbs for authentic virus after the third immunization. **d-e** the number of IL-2 (**d**) and IFN-γ (**e**) secreting cells in each group after stimulated (n=3-4). **f** Body temperature change after infection in each group(n=3-4). **g** Nasal swab viral load after infection in each group (n=3-4). **h** Throat swab viral load after infection in each group (n=3-4). Data were presented as mean±SD.

**Supplementary information, Fig. S14**


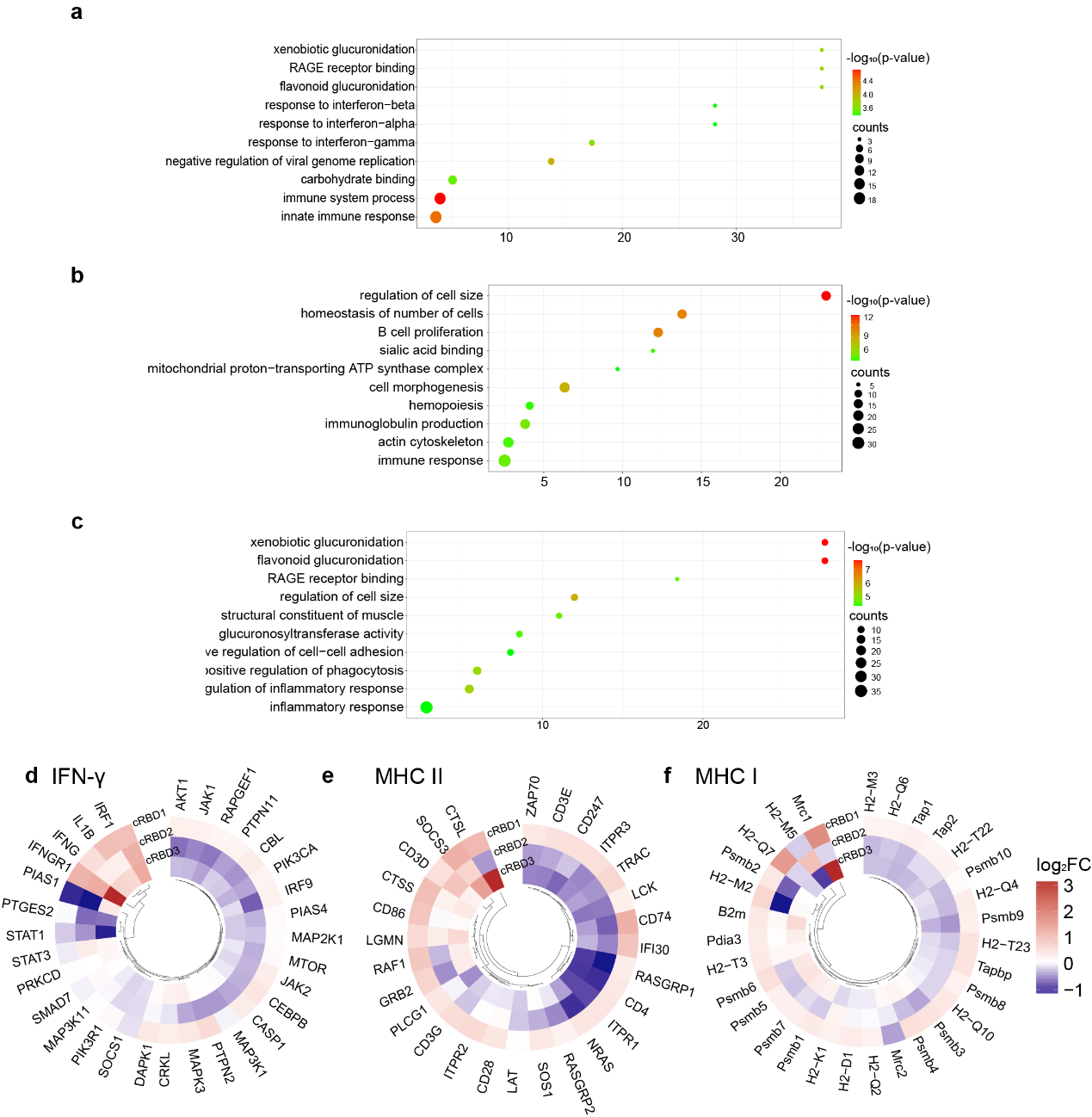


**Supplementary information, Fig. S14** **Analysis of mRNA sequencing results. a-c** Top ten most significant terms of Go enrichment analysis of DEGs (p<0.05, |log_2_FC|>1) in cRBD1(**a**), cRBD2(**b**), cRBD3(**c**). **d** Expression fold change of IFN-γ pathway-associated genes in the groups relative to the NC group. **e** Expression fold change of MHCII pathway-associated genes in the groups relative to the NC group. **f** Expression fold change of MHCII pathway-associated genes in the groups relative to the NC (negative control) group.
